# Clinical and Behavioral Determinants of Type 2 Diabetes Remission After Bariatric Surgery: An Explainable Machine Learning Approach

**DOI:** 10.3390/jcm15176542

**Published:** 2026-08-24

**Authors:** Metab Algeffari, Haifa F. Alhasson, Shuaa S. Alharbi

**Affiliations:** 1 Department of Family and Community Medicine, College of Medicine, Qassim University, Buraydah 52571, Saudi Arabia; m.geffari@qu.edu.sa; 2 Abdullah Al-Othaim Diabetes Center, Medical City, Qassim University, Buraydah 52571, Saudi Arabia; 3 Department of Information Technology, College of Computer, Qassim University, Buraydah 52571, Saudi Arabia; hhson@qu.edu.sa

**Keywords:** artificial intelligence, bariatric surgery, diabetes remission, integrated diabetes care, machine learning, metabolic complications, risk stratification, type 2 diabetes mellitus

## Abstract

**Background**: Achieving remission of type 2 diabetes mellitus (T2DM) after bariatric surgery represents a critical opportunity to reduce long-term diabetes-related complications, including cardiovascular disease, nephropathy, neuropathy, and retinopathy. However, remission rates vary widely across patients, and identifying modifiable clinical and behavioral determinants remains essential for optimizing integrated metabolic care. **Objectives**: In the current study, we aimed to (1) classify type 2 diabetes mellitus (T2DM) remission status after bariatric surgery through clinical, anthropometric, and behavioral variables at follow-up; (2) identify the main model-based determinants of remission status and explainable machine learning using the preoperative model for baseline risk stratification with surgical candidates. **Methods**: We performed a retrospective cross-sectional study on 233 patients with T2DM who had bariatric surgery at a tertiary referral center. We made use of two analytical frameworks: a full-feature approach to identify the current remission status in a cross-sectional manner and a preoperative approach to make a temporal classification of the baseline for the first time. We trained and internally assessed 14 machine learning and deep learning classifiers. We evaluated model interpretability using SHAP. **Results**: In the full-feature cross-sectional classification, the Bottleneck Network performed best (ROC AUC = 0.889). SHAP data identified percentage weight regain, pre- and post-surgical body mass index, HbA1c, and oral hypoglycemic agent use as the dominant model-associated factors. In the restricted preoperative setting, the Extra Trees model achieved an AUC of 0.707, which is a lower level but still represents good baseline risk stratification performance. **Conclusions**: The findings indicate that remission status after bariatric surgery is both clinical as well as behavioral, but the post-operative or contemporaneously assessed variables should be looked at as classification (as opposed to prediction) models. The preoperative model may be able to be used in risk stratification, but clinical validation and prospective evaluation should be made prior to clinical implementation.

## 1. Introduction

Type 2 diabetes mellitus (T2DM) is a chronic progressive systemic metabolic disorder of epidemic proportions worldwide. Its incidence is increasing because of rising obesity, sedentary lifestyles, and unhealthy eating behaviors. Worldwide, the estimated number of adults with diabetes was 537.8 million in 2021 and is predicted to rise to 783 million by 2045 [1]. T2DM is associated with a variety of devastating complications including cardiovascular disease, neuropathy, retinopathy and chronic kidney disease, which account for significantly higher levels of morbidity, mortality and health-related cost burden [2,3].

Benefits from bariatric surgery as a highly effective treatment of obesity and T2DM, not only achieving weight loss but also potentially achieving glycemic remission, have drew increasing attention. Standard weight loss surgeries such as RYGB and sleeve gastrectomy have demonstrated remission rates between 30% and 80% in relation to patient- and surgery-related factors [4,5]. The pathophysiologic mediators of remission are profound weight loss and hormonal changes in addition to improved insulin sensitivity [6,7]. Nevertheless, considering the differences observed, classification tools are necessary to individualize patients who can have better disease outcomes, with the aim of achieving remission.

Classic statistical analysis, such as Logistic Regression, was applied to estimate the remission according to the BMI, baseline HbA1c, and duration of diabetes. For example, the DiaRem score [8] and the Ad-DiaRem score [9] incorporate preoperative factors to classify diabetes resolution. Although of practical importance, these cusp models suffer from using linear relations and for not being able to model the complex range of model-attributed determinants in a high-dimensional space.

Machine learning, deep learning approaches provide many opportunities to circumvent these problems. Nonlinear relationship and interaction between features can be modeled by ML models such as RF, GBM and SVM, which show better classification performance [10,11]. Machine learning in medicine and model interpretability have also been widely discussed [12,13]. On the other hand, deep learning (DL) models (e.g., CNNs [14,15] or bottleneck networks [16]) are powerful tools for high-dimensional data analysis and pattern discovery [17,18]. Indeed, these models have shown their viability for anticipating outcomes in a variety of healthcare tasks such as cardiovascular events, hospital readmissions and diabetes control [14,15]. Nonetheless, the integration of ML/DL into bariatric surgery remains in its early stages and there are few studies on T2DM remission.

A fundamental challenge for the adoption of ML/DL in the clinical domain is that they are “black-boxes,” and therefore their interpretability and trust among clinicians is limited. Interpretable AI (XAI) methods, like SHapley Additive exPlanations (SHAP) and Local Interpretable Model-agnostic Explanations (LIME), have been introduced to address this problem. These methods offer interpretability by quantifying the influence of features on model classification s, thus making it simpler for clinicians to comprehend and trust the outputs [16,17]. Regarding bariatric surgery, XAI may reveal important model-attributed determinants of outcomes, such as weight regain, preoperative HbA1c values, and adherence to dietary recommendations, thus facilitating personalized medicine and joint decision-making [18].

To fill these gaps, the present study aims to develop and evaluate explainable machine learning and deep learning models to classify T2DM remission after bariatric surgery. In particular, the purpose of this study is to answer the following research questions:

RQ1: How can ML and DL models classify T2DM remission after bariatric surgery using preoperative clinical, metabolic, and behavioral variables?

RQ2: Which preoperative features are the most influential model-attributed determinants of T2DM remission, and how do they interact to shape model classifications, as identified through explainable AI techniques like SHAP and LIME?

RQ3: How do the discriminative performance metrics of the explainable ML/DL models developed in this study compare contextually to the published validation performance of traditional clinical scoring systems such as DiaRem and Ad-DiaRem, and what are the relative advantages of each approach in terms of feature scope and interpretability?

There is a crucial distinction to this study and we want to emphasize from the outset that, since the classifier and outcome variables were measured at the same time through a single cross-sectional interview (Section 3.1), models with postoperative variables that were measured simultaneously with remission status (e.g., percentage weight regain, current BMI, current HbA1c) are classification models of current remission status, not classification models of future remission. Only the subset of models that were measured only before or independently of the outcome (Framework B) are a time-dependent classification model. We will keep this distinction throughout the Methods, Results, and Discussion.

Based on our review of the literature and the multifactorial nature of T2DM remission, we hypothesize that:

**H1.**  *The best-performing ML/DL model will have a statistically significantly higher ROC AUC than the DiaRem (published AUC range: 0.67–0.77) and Ad-DiaRem (published AUC range: 0.74–0.83) clinical scoring systems as measured by the DeLong test at α = 0.05.*

**H2.**  *SHAP-based explainability analysis will identify at least one behavioral variable (e.g., meal frequency or snacking habits) among the top five most important model-attributed determinants in the best-performing model, in addition to the conventional metabolic and anthropometric features used in standard scoring tools.*

**H3.**  *The top five SHAP-ranked features will have ≥60% overlap (Jaccard similarity ≥ 0.60) among the three best-performing models. This indicates consistency and generalizability of the explainability framework.*

The top-five and Jaccard ≥ 0.60 thresholds used in H2 and H3 were chosen as a pragmatic, pre-specified criteria for this exploratory study rather than the statistical cutoffs. In Section 4.4, we report the exact rankings and similarity values, so that readers can assess the underlying evidence independently of the threshold chosen.

To be clear, the main goal of this study is clinical: to characterize the metabolic and behavioral phenotype of T2DM remission following bariatric surgery and identify which preoperative factors can meaningfully stratify future surgical candidates by likely remission probability. The comparison of 14 machine learning and deep learning algorithms is undertaken for this clinical objective rather than as a methodological contribution to the machine learning literature.

In the present study, not all analytical frameworks are the same. Since several of the variables in the full feature analysis were measured contemporaneously with remission status at follow-up, this framework is aimed at cross-sectional classification of current remission status, not predicting future remission. In order to address the clinically relevant question of baseline risk stratification, we also investigated a restricted preoperative framework, where only variables available before surgery were considered. Thus, this study had two goals that were related but not identical, namely explanation of current remission status and preoperative classification of surgical candidate risk stratification.

## 2. Related Work

### 2.1. Bariatric Surgery and Diabetes Remission

Bariatric surgery is widely established as one of the most successful treatments for obesity and T2DM with sustained benefits beyond weight reduction. There is clinical evidence to suggest it is also able to drive the remission of diabetes by mechanisms involving enhanced GLP-1 secretion, enhanced insulin sensitivity, and alterations of gut physiology [4,5]. While effectiveness is established, remission rates differ significantly according to the baseline BMI, duration of diabetes, age, and weight regain [6,7].

### 2.2. Machine Learning in Diabetes Prediction and Classification

Machine learning is a promising framework for predicting and classifying human outcomes in diabetes care. ML approaches such as Random Forest, Support Vector Machine and Gradient Boosting models are particularly useful to identify more complex patterns among model-dependent determinants and have been used to predict glycemic control, weight loss and complications. Random Forests, introduced by Breiman [10], are able to model nonlinear relationships between predictors, and SHAP, proposed by Lundberg and Lee [11], can assist in understanding complex model outputs by estimating the contribution of each feature. But although ML can improve classification performance, the interpretability of the results is still a critical challenge in clinical decision-making.

### 2.3. Deep Learning in Healthcare

Deep learning techniques such as convolutional networks (CNNs), recurrent neural networks (RNNs), and bottleneck networks are especially suitable for analysis of high-dimensional data. These models have been utilized to forecast clinical outcomes across diverse healthcare areas such as cardiovascular disease [12], hospital readmissions [13] and diabetes remission [14,15]. Despite the promise of DL models, they are largely unutilized in performing bariatric surgery, in part because of their substantial data requirements, the need for extensive external validation, concerns regarding interpretability, and the limited integration of such systems into real-time surgical workflows.

### 2.4. Explainable AI in Clinical Decision-Making

Methods such as interpretable AI (XAI) have recently received attention in healthcare as an approach to solve the interpretation challenges of ML/DL models. This lack of transparency makes it difficult for clinicians to trust model classifications and to incorporate them into clinical decision-making. Healthcare institutions are often reluctant to adopt opaque models and instead prefer approaches that offer interpretable and justifiable outputs. SHAP and LIME quantify the contribution of individual features to model classifications, thereby enhancing transparency and clinical trust [16,17]. In the context of bariatric surgery, XAI methods can identify dominant model-attributed determinants of outcomes, such as weight regain, HbA1c levels, and dietary adherence, thereby supporting clinicians in evidence-based decision-making [18].

Although advancements towards classification of diabetes remission have occurred, there continue to be areas of unmet need. Most have been performed on small, usually very homogenous samples, making it difficult to generalize these findings. Yet behavioral and psychosocial factors have been shown to impact remission and are not fully represented here. Furthermore, the use of XAI in bariatric surgery is nascent. This study is aimed at filling those gaps, through building interpretable ML and DL models focused on SHAP-based explanations for the most predictive factors of remission.

## 3. Materials and Methods

### 3.1. Study Design and Model Overview

This study used a retrospective cross-sectional design. Data were collected from January 2020 to April 2024 from patients who had previously undergone bariatric surgery at King Fahad Specialist Hospital. The time since surgery was different among the participants (e.g., the ‘years since surgery’ variable). Both predictor variables and remission status were determined at the same time by phone interview and medical record review. T2DM remission was defined according to the 2021 ADA consensus statement [19] as HbA1c < 6.5% for at least 3 months after discontinuation of all glucose-lowering medications. In this cohort, 147 of 233 patients (63.1%) achieved remission. Therefore, the models developed in this study categorize current T2DM remission status based on pre-operative historical variables and clinical and behavioral characteristics and do not predict the future from previous pre-operative data alone.

Out of 251 patients that were initially screened as having undergone bariatric surgery with a diagnosis of T2DM, 18 patients (7.2%) were excluded due to missing laboratory or anthropometric data, most notably missing HbA1c measurements due to missed follow-up appointments. The other 233 patients who were provided with all 15 study variables were the final data set. The initial study cohort comprised 233 patients. We used a complete case analysis approach. Eighteen patients (7.2%) of the initially screened cohort were excluded due to incomplete records. To assess the potential for selection bias, we compared available baseline characteristics among excluded and included patients (age, sex, surgery type) and found there were no statistically significant differences (all *p* > 0.05). Nevertheless, the possibility of residual selection bias cannot be completely excluded.

Since post-operative features (i.e., weight, post-surgery BMI, %WR, %EWL, %TWL, current A1c, meal frequency, snack frequency) are measured simultaneously with the remission label, the full-feature models are not classifications but cross-sectional classification. This approach is likely to be useful for the clinical study, if the modifiable post-operative factors are most influential in distinguishing patients who are in remission from those who are not, and it can be used to identify better treatment for these patients in the future. However, this design cannot establish temporal precedence or causality. Future research should adopt a longitudinal design, in which only pre-operative variables are considered, and post-operative remission is evaluated at a fixed follow-up time interval.

This cross-sectional study was conducted at King Fahad Specialist Hospital (KFSH) between January 2020 and April 2024. KFSH is a tertiary referral center specializing in metabolic and bariatric surgery and manages a high volume of patients undergoing surgical treatment for obesity-related comorbidities, including type 2 diabetes mellitus (T2DM). The study evaluated the ability of machine learning and deep learning models to classify T2DM remission after bariatric surgery. Eligible participants were patients with T2DM who had undergone bariatric surgery and had available follow-up data relevant to remission assessment. Patients with incomplete data or missing critical variables were excluded from the analysis. Of the initially screened patients, 233 met the inclusion criteria and were retained in the final study cohort after application of the exclusion criteria. To identify the most informative predictive strategy, 14 classification models were evaluated in the study:

- Machine learning (ML) methods: Random Forest, Gradient Boosting, Support Vector Machine, Logistic Regression, Extra Trees, AdaBoost, K-Nearest Neighbors, Naïve Bayes, Decision Tree, and Linear Discriminant Analysis.
- Deep learning (DL) architecture: Bottleneck Network, Very Deep Network, Pyramid Network, and Wide Network. All these models were trained on a structured dataset incorporating anthropometric, clinical, weight loss, and lifestyle biomarkers. The model performance was measured in five critical evaluation metrics: accuracy, ROC AUC, precision, recall, and F1-score, providing a valid and balanced comparison of all models on type 2 diabetes remission after surgery.

#### Analytical Frameworks

We developed two complementary approaches for the clinical study to address the temporal limitations of our cross-sectional design.

Framework A (Cross-Sectional Classification—Full Feature Set): All 15 features (Table 1, Features 1–15) were used as inputs. Since eight of these features are measured simultaneously with the remission outcome, this framework is not prospective for prediction. Its primary aim is to characterize the metabolic and behavioral phenotype of remission and to identify modifiable post-operative factors (i.e., dietary adherence and weight trajectory) that are most likely to distinguish patients in remission from patients not in remission. These findings can inform targeted post-operative interventions and care planning.

Framework B (Preoperative Classification—Restricted Feature Set): Only seven features were measured prior to or independently of the outcome (Table 1, Features 1–7: age, height, pre-surgery weight, pre-surgery BMI, A1c before surgery, years since surgery, OHA status) were considered. This framework is a temporally valid prediction model with all model-attributed determinants prior to the outcome and the only framework from which we can make future clinical decision-making conclusions. However, meal frequency and snack frequency were measured at the same time as the outcome (in the follow-up phone interview) and therefore were not evaluated in Framework B, even if they are associated with preoperative counseling. In the Results and Discussion, findings are attributed to one of the two frameworks to avoid the conflation of cross-sectional association with future prediction.

We distinguish between two modeling paradigms with different assumptions and clinical explanations. Framework A is an explanatory (cross-sectional classification) model, since eight of its fifteen input features are measured at the same time as the outcome to characterize the metabolic and behavioral profile of the patient in remission. Framework B is a prognostic (temporally valid prediction) model: as all seven input features are measured prior to or independently of the outcome, it is able to estimate the probability of a future outcome from antecedent information.

The present study used two very complementary frameworks with different objectives. Framework A took into account the complete set of preoperative and follow-up variables that were collected before the beginning of the trial and therefore was meant to be used in cross-sectional classification of the current remission status. Framework B only had preoperative data available before surgery and was designed to test the temporal significance of preoperative prediction for the purposes of baseline risk stratification. We point out that variables such as current HbA1c, postoperative body mass index, and percentage weight regain are not temporally appropriate for true preoperative prognostic modeling at all and should be treated as a classification framework, not a prediction framework.

### 3.2. Data Collection and Preprocessing

The data were retrieved from medical records of patients who had undergone bariatric surgery at King Fahad Specialist Hospital. As part of routine clinical follow-up, the clinical care team conducted structured telephone interviews between January 2020 and April 2024 to assess current weight, dietary habits, medication status, and HbA1c values. The de-identified data from these clinical encounters and related medical records were subsequently used for research purposes. No additional patient contact was initiated for this study.

#### 3.2.1. Dataset Overview

This study involved the use of a dedicated diabetes remission dataset that included patient information from patients undergoing bariatric surgery. This study involved the use of a dedicated diabetes remission dataset that included patient information from patients undergoing bariatric surgery. The final cohort comprised 233 patients, of whom 147 (63.1%) achieved T2DM remission and 86 (36.9%) did not. A total of 15 key features were extracted, which contained a blend of physiological measures, metabolic indexes, and behavioral determinants. Those aspects offered a holistic knowledge of every medical patient’s characteristic and ability to obtain remission of T2DM (type 2 diabetes mellitus) after operation. Table S1 in the Supplementary File summarizes the baseline and postoperative characteristics of the patients stratified by T2DM remission status. Of these, 147 (63.1%) achieved remission and 86 (36.9%) did not. The cleaned and finalized dataset used for model development and analysis is provided in Supplementary Material S2: DM_remission. The baseline and postoperative characteristics of the study cohort stratified by remission status are presented in Table S1 (Supplementary Material S1).

#### 3.2.2. Feature Variables

The data represented a wide variety of data with five core categories. Anthropometric data comprised height (cm), pre-surgery weight (kg), current weight (kg), pre-surgery BMI (kg/m^2^), and post-surgery BMI (kg/m^2^). Weight loss indices captured percent weight reduction (%WR), percent excess weight loss (%EWL) and percent total weight loss (%TWL), giving a measure of the extent of the weight loss after bariatric surgery. The clinical parameters included measured hemoglobin A1c level taken before surgery and in the follow-up, as well as the status of use of oral hypoglycemic agents (OHAs). Long-term outcomes were evaluated with temporal and demographic characteristics including patients’ age and years post-op. Finally, lifestyle indicators were recorded through the mean number of meals and snacks consumed per day, consistent with post-intervention behavior.

As shown in Table 1, 8 of the 15 features are measured simultaneously with the remission outcome. Therefore, the full-feature models should be seen as cross-sectional classification models that characterize the metabolic and behavioral profile associated with remission, not as prediction models. The clinical benefit of this approach is to identify modifiable post-operative factors that most strongly differentiate patients who achieve remission from those who do not, resulting in targeted interventions.

#### 3.2.3. Data Preprocessing Steps

To ensure data quality and readiness for machine and deep learning model development, the following preprocessing steps were applied. The missing values for key variables (such as A1c measurements, weight-related metrics) were removed to preserve the data. Since imputation methods can lead to biases, we only kept complete cases. We did not perform multiple imputation to avoid model-based assumptions in a relatively small sample. This is still a limitation, and future studies should test this with sensitivity analyses. Then, we standardized continuous variables with StandardScaler to maintain similarity throughout numerical features. This step has enabled attributes such as weight and BMI, which naturally differ in scale and units, to be evenly weighed and considered in model training. We converted the target variable of remission status of type 2 diabetes mellitus (T2DM) to binary in the form of 0 for no remission and 1 for successful remission. This encoding simplified the interpretability of the models and could work well with binary classification. The number of excluded cases due to missing data was small (*n* = 18, 7.2% of the initial screening cohort). Missing laboratory data (A1c measurements) was due to patients not being able to meet scheduled follow-up appointments. We compared the demographic characteristics (age, sex, surgery type) of the excluded and included patients and found no statistically significant differences (all *p* > 0.05) and concluded that missingness was like MCAR in this sample. However, this is a limitation, and further studies should be performed with multiple imputation or sensitivity analyses to assess the strength of the findings when different missing data assumptions are used.

Lastly, the dataset was further split into training and testing sets via stratified sampling. We preserved remission and non-remission cases in both sets of samples, thus maintaining the fair evaluation of the model and attenuating the effects of class imbalance. Specifically, a 70/20/10 stratified split was applied, generating 163 samples for training (103 remission, 60 non-remission), 47 samples for validation (29 remission, 18 non-remission), and 23 samples for testing (15 remission, 8 non-remission). The validation set was used to tune the hyperparameters and stop the deep learning models during training, while the test set was reserved for final evaluation. With 15 input features and 233 total samples, the feature-to-sample ratio is roughly 1:15.5. The overall ratio is acceptable for the machine learning models employed in this study.

#### 3.2.4. Definition of T2DM Remission

Remission is defined as a hemoglobin A1c (HbA1c) below 6.5% (48 mmol/mol) at least 3 months after all glucose-lowering drugs (including oral hypoglycemic agents and insulin) had been discontinued. Non-remission was defined as when the patient had an HbA1c ≥ 6.5% and had glucose-lowering medication still in place at the time of the measurement. The patients’ status of remission at the onset of the data collection season (January–April 2024) was determined by medical record review of the most recent HbA1c (15 days before the diagnosis) and telephone interview confirming medication status. The same criteria were used for all 233 patients in the study sample. The time since surgery passed among participants and was measured by the “years since surgery” variable that was used as a predictor in the models.

The OHA status (current use of oral hypoglycemic agents) was used as a predictor variable in the classification models. As medication cessation is part of the ADA remission definition, this brings a degree of overlap between a predictor and the outcome. We have considered OHA status in the classification models because it gives us important information about the progression of the disease, pharmacological dependence, and β-cell reserve beyond the binary remission label. We will note that this partial circularity may make it too much of an issue that OHA status may be misleading, and future studies should be done in which we will test the performance of the models if OHA status is absent in order to ensure that the classification is not redundant.

### 3.3. Overview of Machine Learning and Deep Learning Models

#### 3.3.1. Machine Learning Models

This study utilized 14 classification models—10 machine learning (ML) algorithms and 4 deep learning (DL) architectures—to classify type 2 diabetes mellitus (T2DM) remission after bariatric surgery. These models vary in complexity, interpretability, and computational requirements. Ensemble methods such as Random Forest (RF), Gradient Boosting (GB), Extra Trees (ET), and AdaBoost leverage multiple weak or strong learners to improve predictive accuracy, with RF and GB particularly excelling in handling non-linear relationships, although they may require substantial computational resources or careful parameter tuning. Classical ML models, including Support Vector Machine (SVM), Logistic Regression (LR), Linear Discriminant Analysis (LDA), Naïve Bayes (NB), Decision Tree (DT), and K-Nearest Neighbors (KNN), offer advantages in interpretability, simplicity, or efficiency. SVM performs well in high-dimensional and non-linear spaces but can be computationally demanding, while LR and LDA are efficient for linearly separable data. NB provides fast, scalable performance under the assumption of feature independence, whereas DTs, although easy to interpret, are prone to overfitting. KNN is intuitive and effective for small datasets but suffers in high-dimensional settings and can be computationally expensive. Table 2 presents a consolidated summary of these models and their defining characteristics.

The 14 classifiers were chosen to cover a range of modeling assumptions relevant to this clinical problem: interpretable linear baselines (Logistic Regression, LDA) to test approximate linear separability; ensemble tree-based methods (Random Forest, Extra Trees, Gradient Boosting, AdaBoost, Decision Tree) to capture nonlinear interactions while maintaining feature-importance interpretability; instance-based and probabilistic baselines (KNN, Naïve Bayes); a margin-based classifier (SVM); and four deep learning models to observe whether representation learning has any advantage over classical approaches in this small-sample situation. This range is chosen to directly answer RQ1 by identifying which class of modeling assumptions best suits this clinical classification problem instead of trying to benchmark all classifiers in a random way.

#### 3.3.2. Deep Learning Models

This study also incorporated four deep learning architectures, each offering distinct representational strengths. The Bottleneck Network uses a narrow-hidden layer to transform input data into a reduced representation, which makes it useful for feature extraction and dimensionality reduction. However, it requires careful tuning to prevent underfitting. The Very Deep Network allows for the use of many stacked hidden layers to learn more complex hierarchical patterns, but its depth requires large datasets, significant computational resources and strong regularization so as not to overfit. The Pyramid Network gradually increases then decreases neurons in each of the layers to capture the multi-scale features and to keep the model in equilibrium in complexity with tuning but has hyperparameter sensitivity. In comparison, the Wide Network increases the number of neurons in each layer to learn the various feature representations, and although this method is beneficial for learning broad patterns, it is prone to overfitting in the absence of adequate regularization or sufficient training data.

We note that a sample size of 233 patients is small compared to typical deep learning applications. We include 4 neural architectures in the analysis to test if there is any advantage to representation learning in this small sample size and to test dropout, early stopping, and held-out validation to avoid overfitting. The differences between architectures should be taken with care, as they may be a reflection of sampling variance and not architectural superiority.

### 3.4. Experimental Setup

The performance of machine learning/deep learning models is very dependent on careful experimentation and tuning. Hyperparameter tuning and model evaluation strategies to achieve robust and reliable performance are described below. By methodically constructing systems and fine-tuning individual models, the experimental setup is intended to give the best possible outputs by overcoming problems of imbalanced clinical datasets and diverse model complexity. The hyperparameter tuning strategy and evaluation metrics used to assess model performance are detailed in the next subsections.

#### 3.4.1. Hyperparameter Tuning Strategy

In light of the moderate class imbalance (63.1% remission, 36.9% non-remission), class-weighting was used for all classical models and weighted loss functions for deep learning models instead of resampling techniques (e.g., SMOTE) to avoid introducing artificial data into the small sample size. This approach was employed for all 14 models.

These imbalance-mitigation procedures were applied consistently within the model development process to reduce the risk of biased performance estimates.

The optimization of hyperparameters was carried out in a systematic and model-specific mechanism to achieve better results across all the classification models applied in this investigation. Performance tuning strategies differed by the complexity of the model, the size of the space, and the computational cost. For the eight classical machine learning classifiers—Logistic Regression, Support Vector Machine (SVM), Naïve Bayes, AdaBoost, Random Forest, Decision Tree, K-Nearest Neighbors (KNN), and Extra Trees—grid search and randomized search cross-validation was used:

- Grid search was enforced on models with smaller, distinct parameter spaces, such as Logistic Regression, SVM, Naïve Bayes, and AdaBoost.
- For larger, more complex parameter spaces (e.g., Random Forest and Decision Tree), randomized search was selected as a recommendation to minimize computational costs.
- All classical models were trained using 5-fold cross-validation, which resulted in sound tuning and lower overfitting risk.

For the Gradient Boosting classifier, which requires a larger hyperparameter space (learning rate, maximum depth, number of estimators, subsample ratio, and minimum samples per split), we used randomized search with 5-fold cross-validation to efficiently explore the parameter space while minimizing computational cost. This approach samples a fixed number of combinations of parameters from the specified distribution and provides a good balance of search depth and computational cost when models have several interacting hyperparameters.

For deep learning architectures, fine-tuning techniques were set up based on the network type (through manual and automated strategies):

- The Bottleneck Network was tuned through a manual grid search that relies on early stopping on validation loss for the bottleneck layer size, dropout rates, and learning rate.
- The Very Deep Network also used the Keras Tuner framework to perform random search over 30 trials on architectural parameters such as number of layers, number of neurons per layer, dropout rates, and regularization strategies.
- These models were trained with relevant callbacks to avoid overfitting and improve generalization.

This tiered tuning design method also allowed each model (no matter the complexity) to be optimized in a more efficient and unbiased manner across all experiments. In light of the small cohort size, due to the flexibility of deep learning models, several measures were taken to reduce overfitting risk. These included relatively small network architectures, dropout regularization, early stopping based on validation performance, and held-out validation for training. These precautions have been taken to ensure better training stability and to temper the optimism of the model performance estimates, but the results of the deep learning models should still be taken somewhat skeptically as the sample size is small. Full details of the hyperparameter search space, number of search iterations, optimization criterion, and final selected hyperparameters for each of the 14 models are provided in Supplementary Table S2.

#### 3.4.2. Evaluation Metrics

The main statistical performance metrics used to evaluate and compare these machine learning and deep learning models are summarized in Table 3. Five standard metrics were used: accuracy, precision, recall, F1-score, and ROC AUC. Collectively, these metrics give a comprehensive view of the predictive capability of each model—especially for imbalanced clinical outcomes like diabetes remission. Accuracy shows how accurate classifications are overall, while precision and recall provide metrics representing the model’s ability to detect positive cases and keep false positives or false negatives to a minimum. The F1-score provides a balanced measure that combines precision and recall: it becomes valuable in cases when trade-offs exist between the two. Finally, ROC AUC assesses performance across all decision thresholds, telling you how well a model can discriminate against remission and non-remission cases.

Reporting of this study was guided by the TRIPOD-AI extension, and a completed checklist is provided in the Supplementary Materials.

## 4. Results

In this section, the clinical question is not which algorithm is the most appropriate but which factors distinguish patients who achieve diabetes remission from those who do not and how reliably this difference can be established from the available clinical and behavioral data. A technical comparison of fourteen classifiers serves this clinical objective rather than being an end in itself. The following subsections report model discrimination (ROC curve analysis and comparative performance metrics), 10-fold cross-validation stability, and SHAP-based feature importance for the Bottleneck Network, together providing a comprehensive assessment of predictive capability and the underlying drivers of remission outcomes. Results are presented separately for the two analytical frameworks. The first framework analyzes cross-sectional classification of current remission status using the whole feature set including postoperative and follow-up variables. The second framework analyzes preoperative prediction based on only baseline variables available before surgery. Since these frameworks deal with different clinical questions and have different feature sets, the results in each should be seen in their own analytical context rather than as directly interchangeable model comparisons.

Similar to the section Analytical Frameworks, all results reported under Framework A (full-feature models, including the Bottleneck Network and Random Forest) are interpreted as cross-sectional classifications of current remission status. Only results reported under Framework B (Section 4.3.4), based only on preoperative variables, should be interpreted as temporally valid prediction.

**Figure 1:**
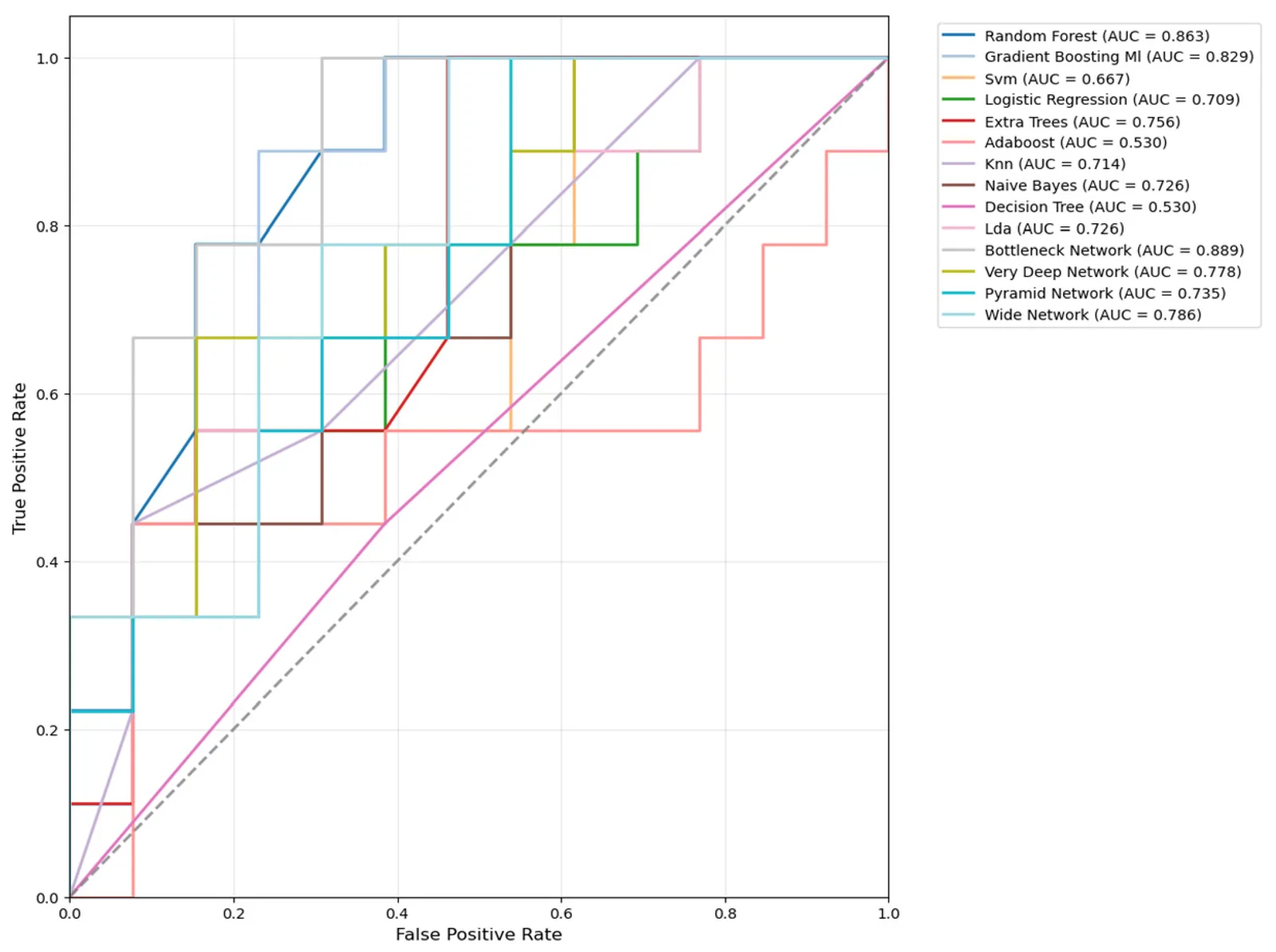
The ROC curves of ML/DL models for classification of type 2 DM remission following bariatric surgery.

**Figure 2:**
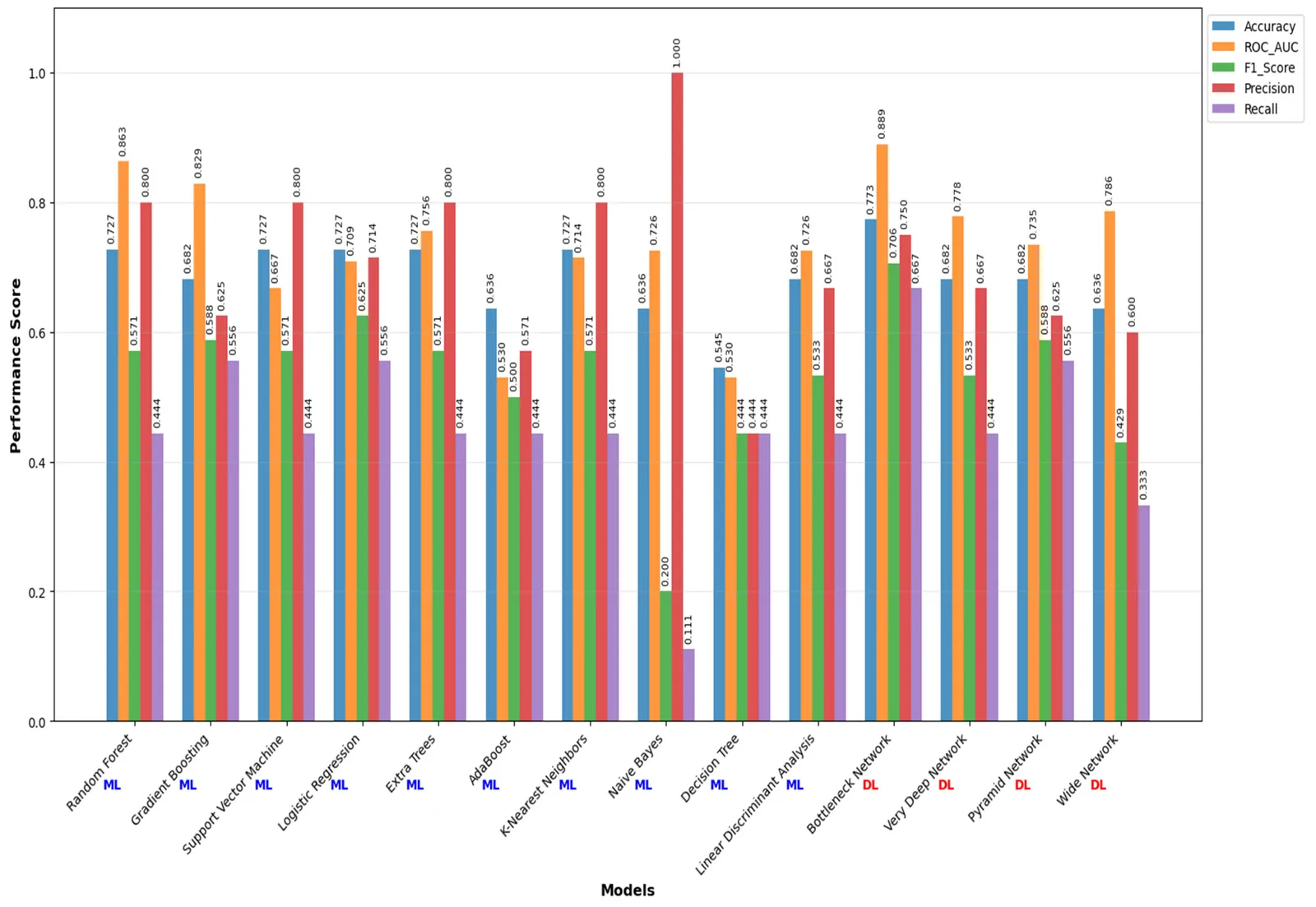
Comparative performance metrics of machine learning and deep learning models in diabetes remission classification with held-out test set (n = 23; train/validation/test split protocol). Note that these results are not directly comparable to the 10-fold cross-validation result presented in Figure 3 that is based on a different validation framework and the full dataset (n = 233).

**Figure 3:**
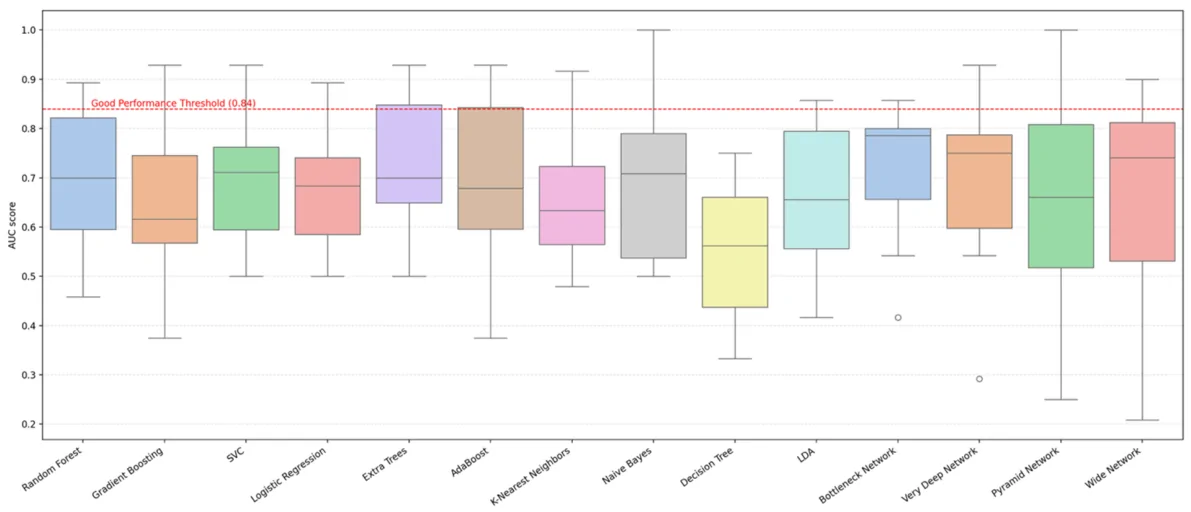
The results of 10-fold cross-validation using ML/DL classification models of diabetes remission classification.

Two complementary evaluation techniques have been applied. First, the final trained models have been evaluated on the set of held-out data (n = 23; 15 remission and 8 non-remission) that is completely absent during training and hyperparameter tuning and is displayed in Figure 1 and Figure 2. Second, we have conducted 10-fold stratified cross-validation on the complete data set (n = 233) to evaluate the stability and generalizability of the discriminative performance of each model across different data sets and present the results in Figure 3. The two approaches serve different purposes: the held-out test set gives us a final unbiased performance estimate under the train/validation/test protocol, while cross-validation quantifies performance variability and reduces dependence on a single random split.

### 4.1. Classification Performance of ML/DL Models (RQ1)

This subsection focuses on RQ1: how can ML and DL models classify T2DM remission after bariatric surgery using preoperative clinical, metabolic, and behavioral variables? The discriminative performance of all 14 models is shown below.

#### 4.1.1. ROC Curve Analysis

Figure 1 presents the Receiver Operating Characteristic (ROC) curves for a suite of machine learning (ML) and deep learning (DL) models employed to classify diabetes remission status. The ROC curve illustrates the trade-off between sensitivity (true positive rate) and specificity (false positive rate) across various classification thresholds, thereby offering a comprehensive assessment of each model’s discriminative capability. The Area Under the Curve (AUC) metric, displayed in the legend, quantifies the overall performance of each model, with values closer to 1.0 indicating superior predictive accuracy.

Bottleneck Network produced the highest AUC (0.889) among the tested models because it learned the underlying remission patterns using a compressed latent representation. Then, the Random Forest model achieved AUC = 0.863, proving its robustness by using ensemble learning and feature bagging for broader generalization. The other deep architectures such as the Wide Network (AUC = 0.786) and Very Deep Network (AUC = 0.778) also achieved competitive performance, with a very clear representation of deep learning models’ ability to capture complex and nonlinear relationships common in clinical remission data. Medically, these results further affirm the role of specific clinical indicators, such as weight regain, baseline HbA1c, and dietary guideline adherence from previous research [20,21,22].

While classical classifiers like Gradient Boosting (AUC = 0.829), Logistic Regression (AUC = 0.709), and Support Vector Machine (SVM) (AUC = 0.667) had moderate predictive power, ensemble-based approaches such as Extra Trees (AUC = 0. 0.707) and AdaBoost (AUC = 0.530) showed varied results, with AdaBoost underperforming significantly. The Decision Tree model also provided only a relatively low AUC of 0.530, which shows that it struggles to generalize well on high-dimensional, nonlinear data. Linear models, namely Linear Discriminant Analysis (LDA) and Naïve Bayes with an AUC of 0.726, respectively, achieved similar performance in this respect, but their assumptions of feature independence and linear separability may have limited their predictive scope. The K-Nearest Neighbors (KNN) algorithm (AUC = 0.714) demonstrated limited effectiveness, presumably because of its sensitivity to high-dimensional feature spaces and absence of learned internal structure.

On the held-out test set (n = 23), the Bottleneck Network (AUC = 0.889) and Random Forest (AUC = 0.863) displayed the best discriminative performance. However, with the very small test set size, these point estimates should be treated cautiously alongside the more stable cross-validated results presented in Section 4.1.3. If verified in future prospective studies, this could help clinicians to better stratify patients by linking predictive analytics with clinical characteristics such as %WR and HbA1c. For example, future validated tools based on this approach could potentially identify those at higher risk for non-remission who would benefit from increased behavioral counseling, although this work is still speculative until external validation and clinical utility testing are completed [23,24,25,26]. This emphasizes the relevance of hybrid and deep approaches for translational clinical studies in which a precise and interpretable risk stratification framework is necessary for customized treatment planning.

To avoid mixing results from different validation frameworks, we present the following subsections separately: Section 4.1.2 shows held-out test-set performance (train/validation/test protocol; n = 23) for all 14 models, while Section 4.1.3 shows 10-fold cross-validation performance (n = 233) for the 10 machine learning models. These two performance sets are not directly comparable and should not be interpreted as measuring the same quantity.

#### 4.1.2. Comparative Performance Metrics

In the full-feature cross-sectional classification framework, the Bottleneck Network achieved the highest discriminative performance (ROC AUC = 0.889). Yet, since this model included variables measured immediately prior to remission status, the performance should be interpreted as classification of current status rather than prediction. In the restricted preoperative framework, the Extra Trees model reached an AUC of 0.707. The lower performance of the baseline-only model was expected, but the one with a more temporal context for preoperative risk stratification was the least problematic. Figure 2 provides comprehensive information on the diagnostic performance of multiple machine learning (ML) and deep learning (DL) models implemented for classifying diabetes remission status. The figure presents five essential evaluation statistics—accuracy, ROC AUC, F1-score, precision, and recall—for the 14 models, providing comprehensive information about the classification performance. And, out of all these models, the deep learning model Bottleneck Network performed the best and consistently as to all the metrics, with the highest ROC AUC (0.889) and accuracy (0.773). It held good results in F1-score (0.759), precision (0.706), and recall (0.750). It confirms how the model generalizes with the same precision, sensitivity, and also specificity. Meanwhile, the performance of the Random Forest model reached a competitive level by achieving 0.727 accuracy, 0.863 ROC AUC and a 0.571 F1-score, which indicated that we were able to efficiently capture nonlinear patterns and mixed types of data. In the same vein, Gradient Boosting performed well with a robust ROC AUC of 0.829, but with lower recall (0.444) showing an increased likelihood of false negatives. Extra Trees and Logistic Regression yielded marginal results, with F1-scores of 0.571 and 0.625, respectively, showing reliability with structured clinical data but insufficient capacity to model more subtle interactions.

Model performance was still suboptimal for some of them despite their advantages in the literature. K-Nearest Neighbors (KNN) and Naïve Bayes had lower precision and recall, with KNN being uniquely sensitive to feature scaling and data sparsity. The AdaBoost and the Decision Tree models achieved the weakest performance in most metrics, and Decision Tree had the lowest recall and F1-score, at 0.111 and 0.200, respectively, which indicates the weaknesses associated with the tendency to overfit and poor generalization in this domain.

Among all linear models, the Linear Discriminant Analysis (LDA) exhibited balanced, albeit moderate, performance across multiple metrics, indicating its usefulness in cases with clearly separable classes. Support Vector Machine (SVM) displayed lower ROC AUC (0.667) and recall (0.444), while the precision level is still acceptable. The other three deep learning models’ performance—Very Deep Network, Pyramid Network, and Wide Network—was satisfactory but not superior to the Bottleneck architecture. For example, the Wide Network produced a solid ROC AUC (0.786) and accuracy (0.635), but recall decreased to 0.600, which was indicative of limitations in identifying true positive cases. Overall, these findings underscore the relevance of choosing and tuning the model architecture to conduct clinical classification. Models like Bottleneck Network and Random Forest not only achieve the best accuracy but also possess a good balance between recall and specificity—making them very good for high-stake clinical environments, where detection of remission must be precise.

We only consider the deep learning models with the held-out test set because training (using a separate validation set for early stopping) is not possible with k-fold cross-validation and the machine learning models are evaluated with both. Given the small size of the held-out test set (n = 23), we recommend reading the cross-validated estimates in Section 4.1.3 as a more reliable indicator of our expected generalization performance in this sample.

#### 4.1.3. K-Fold Cross-Validation Results

The comparative evaluation of fourteen classification algorithms using 10-fold stratified cross-validation indicates that models with capacity to capture nonlinear interactions and ensemble diversity generally exhibit superior discriminative performance, as reflected by higher median ROC-AUC and narrower dispersion across folds. As shown in Figure 3, Extra Trees and Random Forest consistently achieve strong AUCs with comparatively stable variability, aligning with their capacity for variance reduction through bagging and randomized feature selection. SVM with class-weighting performs competitively, particularly in recall, suggesting effective separation in the induced margin despite small-sample constraints. Logistic Regression and LDA provide respectable baselines and interpretable decision boundaries but are limited by their linear form; their performance likely reflects partial linear separability augmented by preprocessing. While Naive Bayes appears relatively competitive in ROC-AUC, its precision–recall profile is weaker, indicating that its independence assumptions capture rank-order discrimination but do not always translate to thresholder classification balance.

Neural architectures exhibit heterogeneous behavior, with the Bottleneck Network demonstrating favorable discrimination and stability relative to very deep or wide configurations. This pattern is consistent with the regularizing effect of dimensionality compression, which can mitigate overfitting in small data regimes. Conversely, very deep and wide MLPs show greater fold-to-fold variability, consistent with capacity exceeding sample complexity. The boxplot distributions further underscore the importance of robustness: models with tighter interquartile ranges and fewer extreme outliers are more reliable for deployment. Practically, these findings suggest prioritizing Extra Trees or the bottleneck MLP for balanced discrimination and stability, with SVM as a strong alternative where sensitivity is paramount.

#### 4.1.4. Information Leakage from Outcome-Proximal Variables

To measure the extent to which the full-feature Bottleneck Network (AUC = 0.889) depends on variables measured simultaneously with the outcome, we performed two ablation experiments. In the single-variable ablation, we removed each outcome-proximal predictor individually and retrained the model. Removing %WR had the largest single-variable decrease (AUC = 0.834, ΔAUC = 0.055), then post-surgery BMI (AUC = 0.847, ΔAUC = 0.042), while behavioral variables (meal frequency, snack frequency) were treated individually with relatively small decreases (ΔAUC < 0.012). In the cumulative ablation, we removed all outcome-proximal postoperative variables (the current HbA1c, OHA status, post-surgery BMI, post-surgery weight, %WR, %EWL, %TWL, meal frequency, and snack frequency) and found that the discrimination of the full-feature model has fallen from AUC = 0.889 to AUC = 0.760 (ΔAUC = 0.129). Full single-variable and cumulative ablation results are provided in Supplementary Tables S5 and S6, respectively. We found that by excluding postoperative and outcome-proximal variables such as current HbA1c, postoperative body mass index and percentage weight regain, the discriminative performance was lower than the full feature model. This is not surprising since these variables are closely related to contemporaneous remission status. On the other hand, the restricted framework is more appropriate for preoperative prediction as it does not have temporal leakage from variables measured after surgery.

### 4.2. Identification of Influential Predictors via Explainable AI (RQ2)

In this subsection, we discuss RQ2: which preoperative features are the most influential model-attributed determinants of T2DM remission, and how do they interact to shape model classifications? SHAP-based global and local interpretability analyses are presented for the highest-performing model within the full-feature classification framework. To improve the transparency and clinical interpretability of the Bottleneck Network, a state-of-the-art deep learning model for classifying diabetes remission status, SHAP (SHapley Additive exPlanations) analysis was performed. SHAP facilitates the decomposition of complex models and results in individual feature contributions, enabling both global and local interpretations. Such interpretability is crucial for clinical context, wherein understanding the rationale behind model classifications is critical for integrating AI in evidence-based decision-making.

To check the stability of the SHAP feature rankings, we recomputed the SHAP values of the Bottleneck Network in 200 bootstrap samples of the training data. The top five features are ranked in 70% of the bootstrap resamples, showing moderate stability beyond the one training/test split reported in the main analysis. To assess model robustness, performance was examined across repeated internal resampling procedures. Although the relative ordering of top-performing models was generally stable, some variability in rankings remained, supporting cautious interpretation of any single model as definitively superior.

#### 4.2.1. Global Feature Impact: SHAP Summary Insights

**Figure 4:**
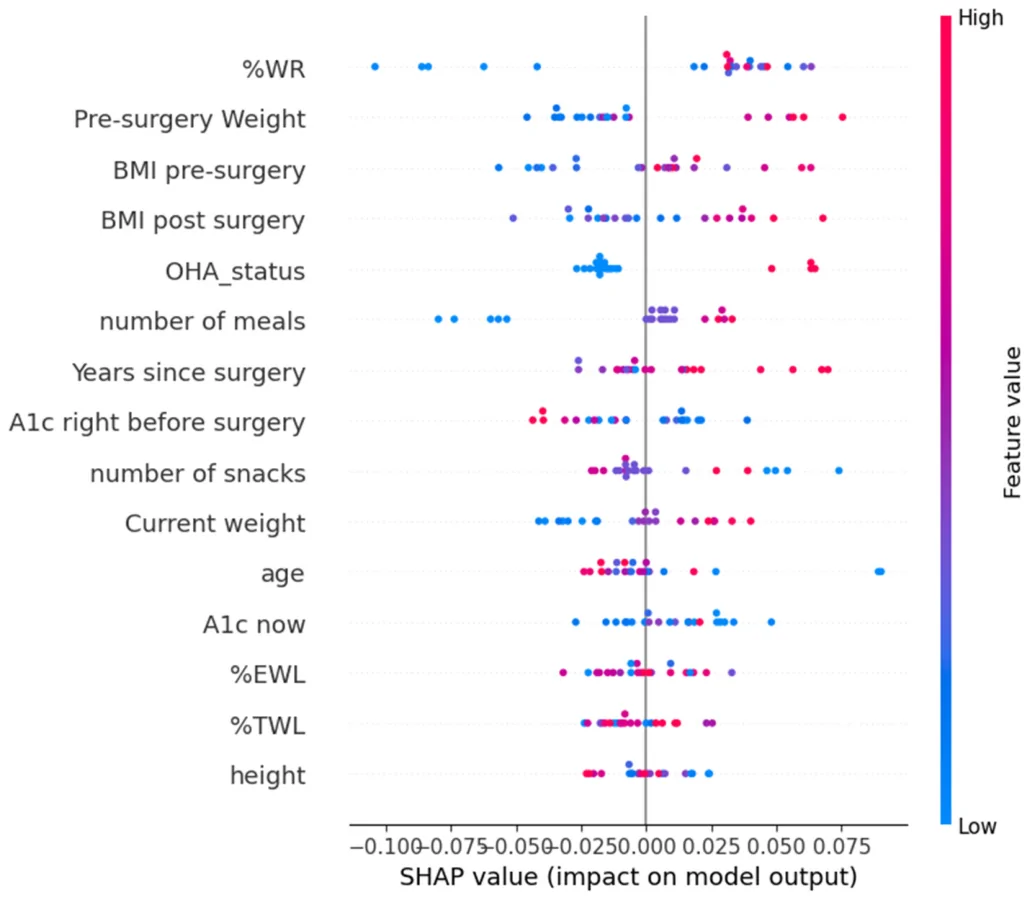
SHAP summary and feature importance plots for the Bottleneck Network model.

Figure 4 displays the distribution and direction of impact based on each feature of each patient case, presented as a SHAP summary plot. The most influential predictor was the percentage of weight regain (%WR), which exhibited the highest magnitude of SHAP values. In clinical settings, evidence is also in line with previous metabolic practice: aberrant post-operative weight regain can, over time, downregulate or overcome the glycemic advantage of bariatric surgery by generating re-insulin resistance, visceral adiposity and β-cell disturbances.

We emphasize that SHAP values indicate the contribution of each feature to the trained model’s output and represent the strength of statistical association of the model. This does not imply causation or the clinical effect size of each factor. The clinical interpretation presented throughout this section is based on the plausible physiological mechanisms based on SHAP-identified associations but should not be interpreted as evidence that the model has demonstrated a causal or quantifiable clinical effect of any given variable on remission.

**Figure 5:**
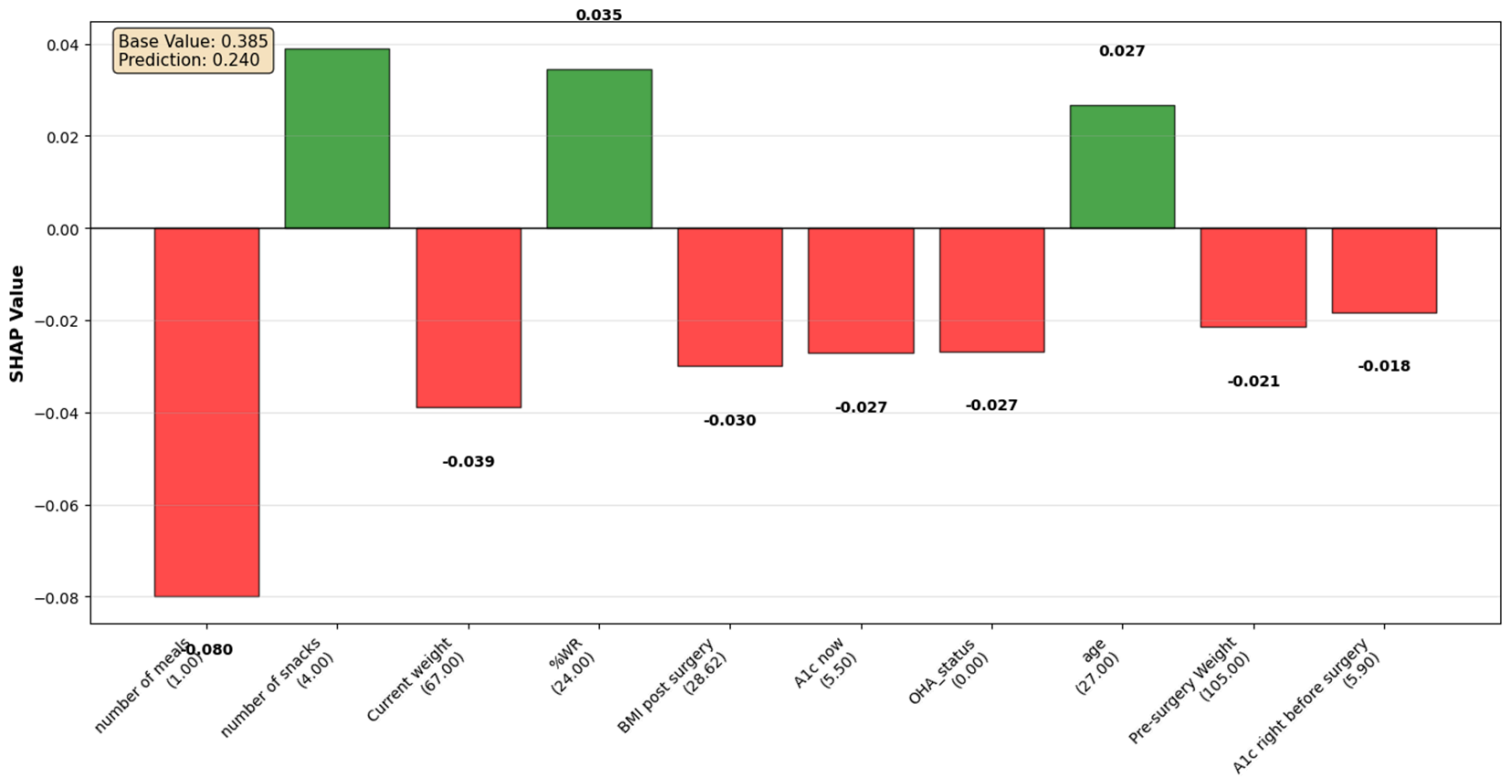
SHAP waterfall plot for representative patient case using Bottleneck Network.

It is important to put these SHAP findings in clinical context. The presence of %WR, BMI and HbA1c as the dominant features is consonant with well-known metabolic principles and not a new clinical discovery. The main benefit of this SHAP analysis is as a model validation mechanism (a model that is logically consistent with clinical knowledge is biologically more likely to be accepted in the clinical setting than one based on correlations that are too weak). Second, and most importantly, the SHAP analysis confirms the relative importance and direction of features at each patient level (Figure 5) in comparison with clinical knowledge and is more robust and consistent with the clinical knowledge that is available. This patient-level decomposition illustrates how, in principle, such models could eventually inform personalized post-operative care plans—though clinical application requires prospective validation and demonstration of improved outcomes over current practice. As we have shown below, behavioral variables (meal frequency and snacking habits) among the top model-attributed determinants are not captured by standard clinical scoring tools such as DiaRem or Ad-DiaRem, highlighting an actionable and underappreciated dimension of post-operative metabolic care.

The pre-surgical and postoperative weight and BMI were also the best features, consistent with evidence of an association between higher baseline BMI and poor glycemic outcomes. These are not simply surrogate values for obesity but instead represent a composite of metabolic burden, systemic inflammation, and long-term insulin resistance, all of which reduce the probability of diabetes remission post-operatively. The value of OHA status (oral hypoglycemic agent usage) was also related to negative SHAP values, especially when the patient remains pharmacologically dependent after surgery. This indicates that persistent OHA use is more likely due to ongoing hyperglycemia or long-standing disease, which are associated with reduced β-cell reserve and diminished remission potential. Other model-attributed determinants including hemoglobin A1c (both current and pre-operative), number of meals, and number of snacks also affected the model’s classifications. Raised A1c levels (particularly before surgery) point to a chronic hyperglycemic state and advanced disease progression, and dietary pattern (such as irregular or excessive snacking) could indicate poor post-operative nutritional adherence, a known barrier to metabolic recovery.

#### 4.2.2. Feature Ranking by Mean SHAP Value: Quantifying Global Importance

To further quantify feature contributions, Figure 4 presents a ranked bar chart of the top fifteen model-attributed determinants based on mean absolute SHAP value. The highest-ranked feature was %WR (mean SHAP = 0.047), followed by pre-surgical weight (0.031) and BMI pre/post-surgery (each 0.026). These metrics are consistent with the literature on obesity-related diabetes, where durable weight loss is a strong independent predictor of remission following metabolic surgery.

Notably, OHA use, number of meals, and A1c before surgery were also prominent. This was indicative of the model’s capacity to consider not only physiological markers (e.g., glucose homeostasis) and behavioral determinants (e.g., eating patterns) of remission. Multidimensionality of this nature is important in clinical work where diabetes remission is in most cases not caused by a single contributor—often, remission of the disease will likely be due to sustained weight loss, glycemic control and adherence to lifestyle treatment and other lifestyle modifications.

We note that a few of the most important SHAP features (%WR, current A1c, and post-surgery BMI) are measured concurrently with remission (Framework A, Table 1). Thus, their high SHAP importance might be due to outcome-proximal or even quasi-tautological values (the current A1c < 6.5% is a part of the remission). The clinical relevance of these features in Framework A is not to provide new model-attributed determinants but to be a target for post-operative interventions: if %WR and dietary behavior are most clearly distinguished from non-remission, we believe post-operative interventions should be more focused on them. For features that are genuinely preoperative and temporally prior to the outcome (pre-surgery BMI, preoperative HbA1c, OHA status), the reader can refer to the results in Framework B (Section 4.3.4, which provide a more conservative and temporally valid interpretation.

#### 4.2.3. Individual-Level Explanation: SHAP Waterfall Plot and Medical Implications

In Figure 5, a SHAP waterfall plot of the sample case with a predicted remission probability of 0.240 versus a model baseline value of 0.385 provides a context for the decision-making aspect of the model at the patient level. So, this patient’s risk factors were poor meal frequency (1/day) (−0.080 SHAP), a high number of snacks (4/day) (−0.039 SHAP) and elevated current weight (67 kg) and percentage weight regain (%WR = 24%) (−0.039 and −0.030 SHAP), respectively. These indicators indicate that there are poor dietary habits and a lack of adequate weight loss in the postoperative period. Further, persisting hyperglycemia (A1c = 5.5%) and subsequent high post-surgical BMI also diminished remission odds. From a clinical perspective, this suggests that glycemic non-remission may be associated not only with residual disease but also with potentially modifiable lifestyle habits like poor dietary maintenance, though causality is not known from this cross-sectional study.

Importantly, there was only slight positive impact, in features such as younger age (27 years) and absence of OHA use, providing additional evidence that although such parameters can provide a promising prognostic value, they are often insufficient to surpass the influence of chronic risk factors. This is consistent with data that describe increased insulin resistance, visceral adiposity, and β-cell dysfunction in postoperative weight regain [21,23]. Behavioral factors, including meal frequency and snacking habits, became important model-attributed determinants, which are like findings that indicated adherence to dietary changes was a potential moderator in maintaining metabolic parameters [24,25].

#### 4.2.4. Clinician Evaluation of SHAP Explainability

As a preliminary usability assessment, five clinicians from a single institution rated the SHAP explanations on seven Likert-scale items with mean scores ranging from 3.60 to 4.60 (Table 4). This small panel size and single-site setup means that the results may only be a hypothesis-generating indicator of clinical acceptability and therefore should be verified with larger clinician panels to draw conclusions regarding clinical adoption. In this limited panel, the clinicians generally felt the SHAP explanations were understandable and potentially helpful for customized post-operative care planning, but this should not be taken as evidence of clinical benefit. Although SHAP rankings were useful in finding important model features, these rankings may be sensitive to sampling variation in a small single-center cohort (hence, SHAP-derived importance patterns should be considered hypothesis-generating and model-specific, not definitive evidence of a stable biological hierarchy). The role of percentage weight regains, BMI, and HbA1c in the SHAP rankings was well suited to clinical experience and established metabolic principles (Q1: 4.60 ± 0.55, Q2: 4.20 ± 0.84; Table 4). Eating frequency and snacking were also selected as behavioral model-attributed determinants based on the SHAP waterfall plots that are not typically included in standard scoring methods such as DiaRem (Q6: 4.40 ± 0.55; Table 4). However, these perceptions reflect hypothetical willingness rather than evidence of clinical benefit, and formal usability studies within actual clinical workflows would be necessary before any implementation. Clinicians also identified limitations. The interpretability scores for the SHAP summary plot (Q3: 3.60 ± 0.89; Table 4) and waterfall plot (Q4: 3.80 ± 0.84; Table 4) were lower, with two clinicians saying the bee-swarm visualization required additional explanation to be understood. One endocrinologist said that without data science training, clinicians might misread the magnitude of the SHAP as the clinical effect size and thus would be more dependent on the data. Moreover, two clinicians indicated that it might be a better idea to use temporal trends (e.g., HbA1c trajectory over multiple follow-up visits) in place of single-point measurements to better serve the patient. These findings suggest that although SHAP-based explanations are generally acceptable and interpretable at the clinical level, formal training and workflow integration would be necessary before application to clinical practice. The full questionnaire items used for clinician evaluation are provided in Supplementary Material S1: Questionnaire Items for Expert Panel.

#### 4.2.5. SHAP for Tree-Based Models

To enable a more direct, methodologically consistent comparison of feature importance across model families, SHAP values were additionally computed for the Random Forest model using TreeSHAP. The top five SHAP-ranked features for Random Forest were %WR, BMI pre-surgery, pre-surgery weight, A1c before surgery, and BMI post-surgery (mean |SHAP| ≈ 0.044, 0.030, 0.029, 0.024, and 0.023, respectively), compared with %WR, pre-surgery weight, BMI pre-surgery, BMI post-surgery, and OHA status for the Bottleneck Network. The two sets shared four features, yielding a Jaccard similarity of 0.67. Because both rankings now use the same underlying attribution method (SHAP), this comparison supersedes the SHAP-versus-Gini comparison originally reported in Section 4.4 (Testing H3) and provides stronger, methodologically consistent evidence regarding feature-ranking consistency across model families.

The SHAP-based interpretability analysis of the Bottleneck Network affirms several foundational clinical truths. First, weight trajectory, particularly post-operative weight regain, is a key factor of diabetes remission. Second, glycemic control pre- and post-operatively, assessed by A1c, is a key metabolic marker. Third, dietary behavior, which often gets lost in the algorithm, emerged as a clinically important part, emphasizing the importance of structured nutritional follow-up in post-bariatric care. These observations confirm the biological plausibility of the model and enhance its translational utility. Being able to explain classifications using medically meaningful variables, such as BMI, %WR, and A1c, in practice means that the model is more trustworthy and applicable for doctors. This is essential when considering the integration of AI into personalized care pathways, both for its transparency and clinical interpretability.

Moreover, the model’s capacity to evaluate both static (e.g., age, baseline weight) and dynamic (e.g., current A1c, number of snacks) features offers a holistic framework for risk stratification framework and therapeutic planning. Patients identified as high-risk non-remission could benefit from more intensive nutritional counseling, pharmacologic optimization, and closer post-operative surveillance.

The SHAP analysis of the Bottleneck Network uncovers that the model’s classifications of diabetes remission follow a clinically consistent structure consisting of a sequence of features. Weight regain, pre-surgical obesity metrics, glycemic indices, and dietary behaviors represent the most powerful determinants, conforming to the prevailing medical framework of metabolic disease evolution and resolution. Furthermore, by adding explainability to deep learning, this exploratory model demonstrated promising discriminative performance and provided interpretable outputs that are consistent with clinical knowledge. However, before being integrated into real-time clinical decision support systems, multi-center external validation, formal calibration and prospective clinical utility studies are necessary.

### 4.3. Comparison with Clinical Scoring Systems (RQ3)

This subsection addresses RQ3: how do the discriminative performance metrics of explainable ML/DL models compare to the published validation performance of standard clinical scoring systems like DiaRem and Ad-DiaRem?

Because the data required for the standard DiaRem and Ad-DiaRem scores were not available, we only reconstructed the scores using the available variables. However, this comparison should be considered exploratory and hypothesis-generating rather than a formal validation or direct head-to-head comparison of these scoring systems.

#### 4.3.1. Bootstrap Confidence Intervals

**Figure 6:**
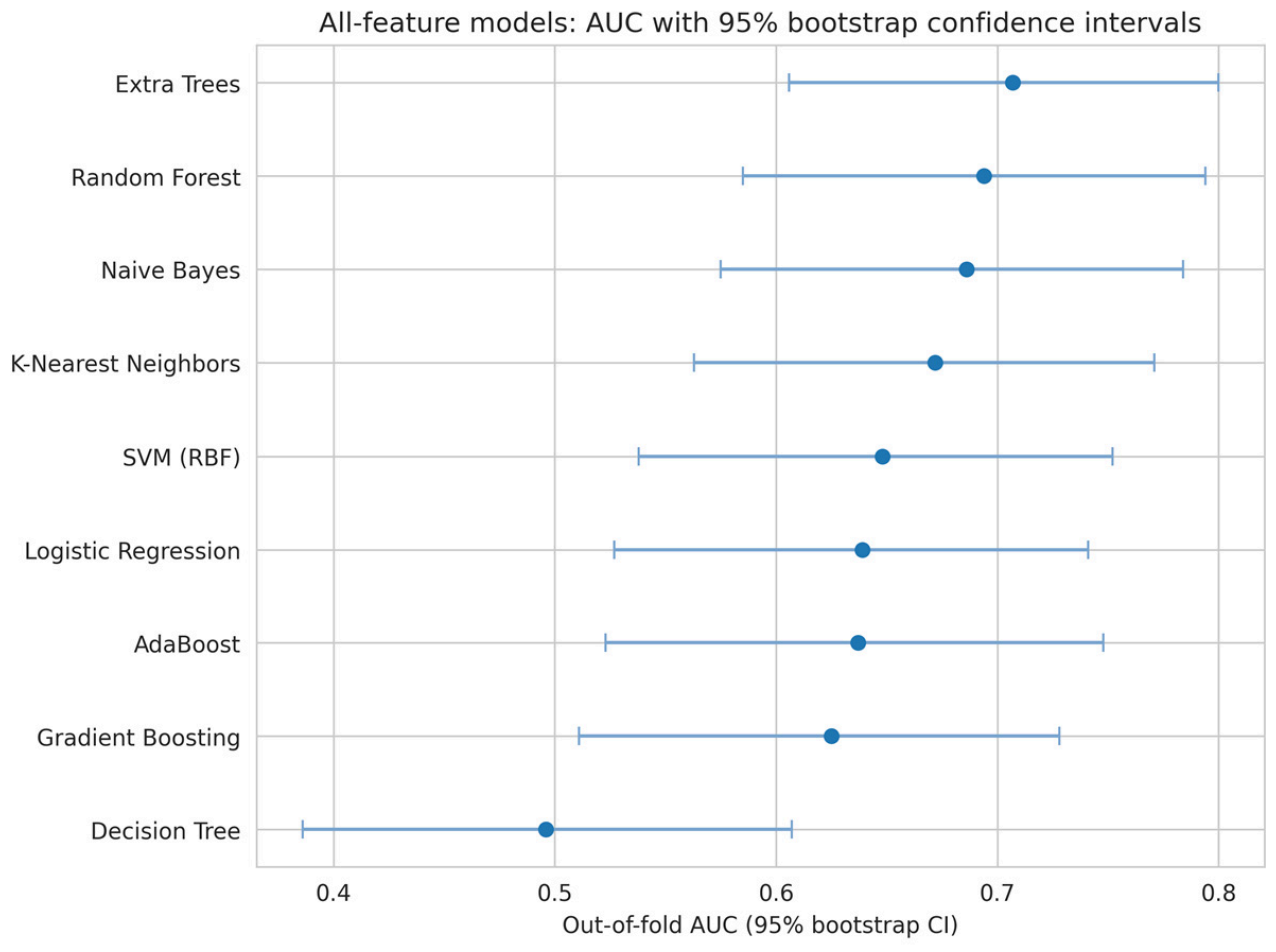
Out-of-fold AUC (95% bootstrap CI) from 10-fold stratified cross-validation (n = 233) for the 10 machine learning models with the full feature set. Filled circles show point estimates; horizontal bars show 95% CIs. Models are ranked by descending AUC. Note that the four deep learning models (Bottleneck, Very Deep, Pyramid, Wide Networks) are not included here because their train/validation/test protocol is not compatible with the out-of-fold cross-validation process used to generate this figure; their held-out test-set performance is reported in Figure 1 and Figure 2.

To assess the model performance beyond standard accuracy, we have also performed several complementary analyses. The AUC with 95% bootstrap confidence intervals (Figure 6) showed that Extra Trees had the best discriminative performance (AUC = 0.707; 95% CI: 0.606–0.800), followed by Random Forest (AUC = 0.694; 95% CI: 0.585–0.794), while Decision Tree had a near-chance performance (AUC = 0.496), highlighting the importance of including confidence intervals when considering differences between models.

We note that the bootstrap confidence interval analysis (Figure 6) was only performed for the 10 machine learning models based on the out-of-fold predictions from the 10-fold cross-validation framework. Four deep learning models (Bottleneck Network, Very Deep Network, Pyramid Network, Wide Network) were not included in this analysis as their training procedure (which uses a separate validation set to start and tune the hyperparameters) is not consistent with the k-fold cross-validation procedure used to make the out-of-fold predictions. Thus, the AUC of 0.707 (Extra Trees) is the best cross-validated ML performance, while the AUC of 0.889 (Bottleneck Network) is the held-out test set performance using the train/validation/test split protocol. Bootstrap 95% confidence intervals for accuracy, precision, recall, F1-score, and Brier score for all 10 cross-validated machine learning models are provided in Supplementary Table S4.

#### 4.3.2. Calibration Curves and Brier Scores

**Figure 7:**
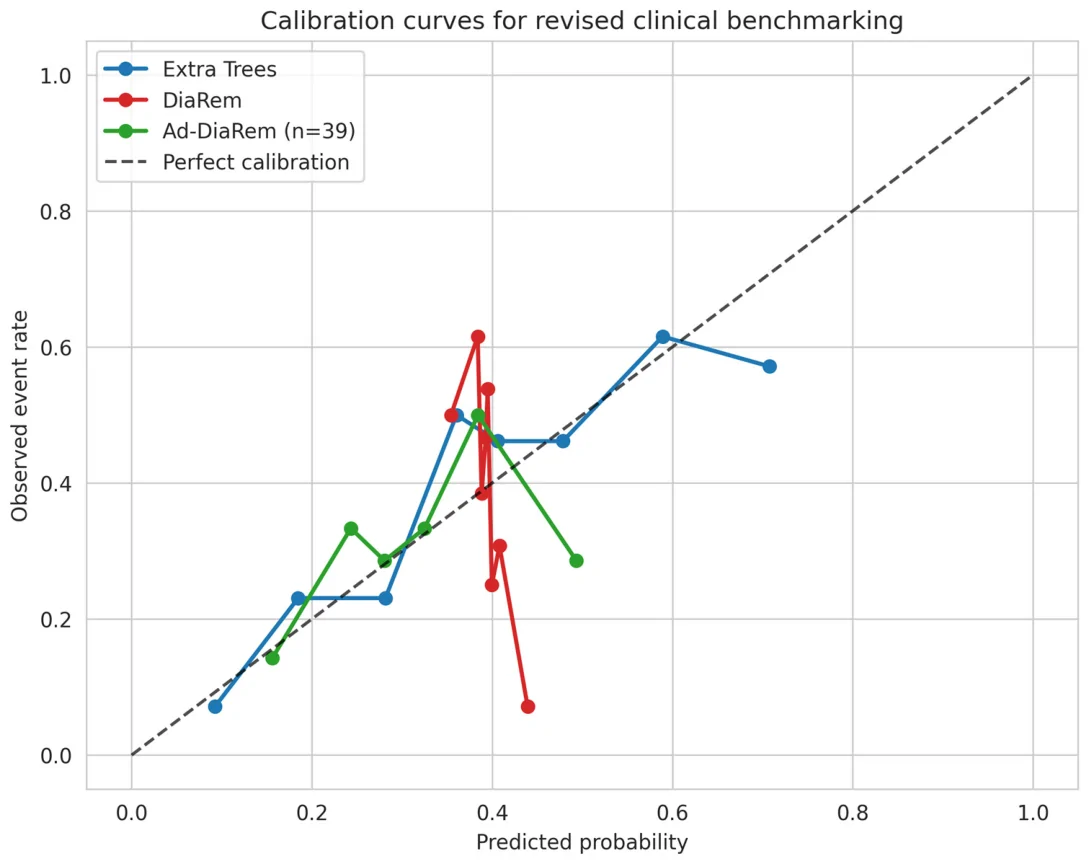
Calibration curves for Extra Trees, DiaRem, and Ad-DiaRem. The dashed line indicates perfect calibration. Ad-DiaRem was computed on a reduced subset (n = 39).

A calibration was performed (Figure 7) in which we found that the best machine learning model (Extra Trees) was closer to the ideal calibration line than the DiaRem and Ad-DiaRem clinical scores, indicating that the predicted remission probabilities more accurately reflect the observed outcome frequencies and can be clinically interpreted.

Quantitative calibration parameters beyond the visual calibration curve, specifically calibration intercept and calibration slope, were not computed in the present analysis. However, Brier score was reported separately as an overall measure of probabilistic accuracy. This limitation is acknowledged and noted as a direction for future work in the section Limitations and Future Directions.

#### 4.3.3. Decision Curve Analysis

**Figure 8:**
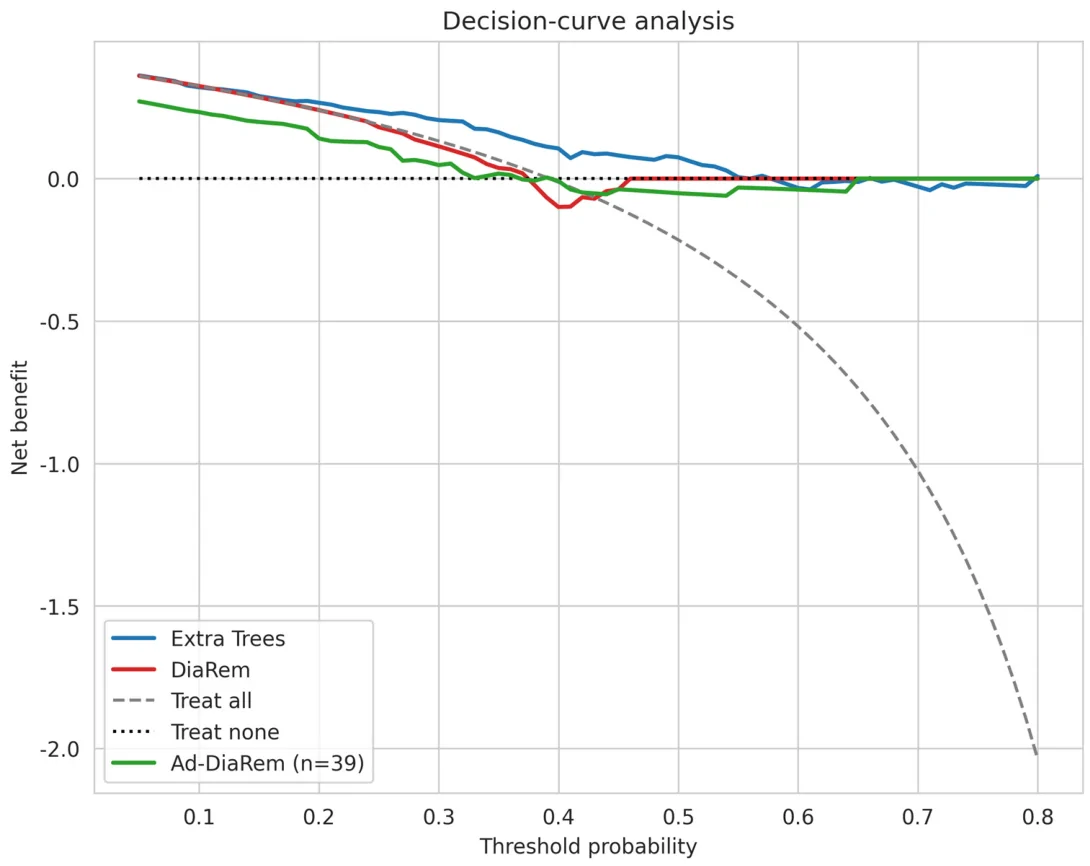
Decision curve analysis of Extra Trees, DiaRem, and Ad-DiaRem (n = 39). The dashed grey line indicates “treat all,” and the dotted black line indicates “treat none.” Higher net benefit reflects greater clinical utility.

From the clinical point of view, probability thresholds should be interpreted based on the use case. Lower thresholds are better when the goal is to select patients who may benefit from closer metabolic monitoring or behavioral support, whereas higher thresholds are better if a more conservative classification strategy is desired. However, these threshold-based interpretations are illustrative only because external validation and calibration should be performed prior to being used in clinical decision-making. Furthermore, our decision curve analysis (Figure 8) discovered that the Extra Trees model has a greater net benefit across a clinically meaningful range of threshold probabilities than the “treat all” and “treat none” reference strategies and DiaRem and Ad-DiaRem benchmark scores. This finding further underscores the potential applications of our model in threshold-based clinical decision-making.

From a clinical perspective, threshold probabilities in the range of 20–80% could correspond to decision points at which clinicians would consider intensifying postoperative behavioral counseling or pharmacological optimization. Within this range, the Extra Trees model provided greater net benefit compared to DiaRem and Ad-DiaRem, suggesting a plausible (but not yet clinically validated) operating range for future prospective evaluation.

#### 4.3.4. Preoperative-Only Model Validity

**Figure 9:**
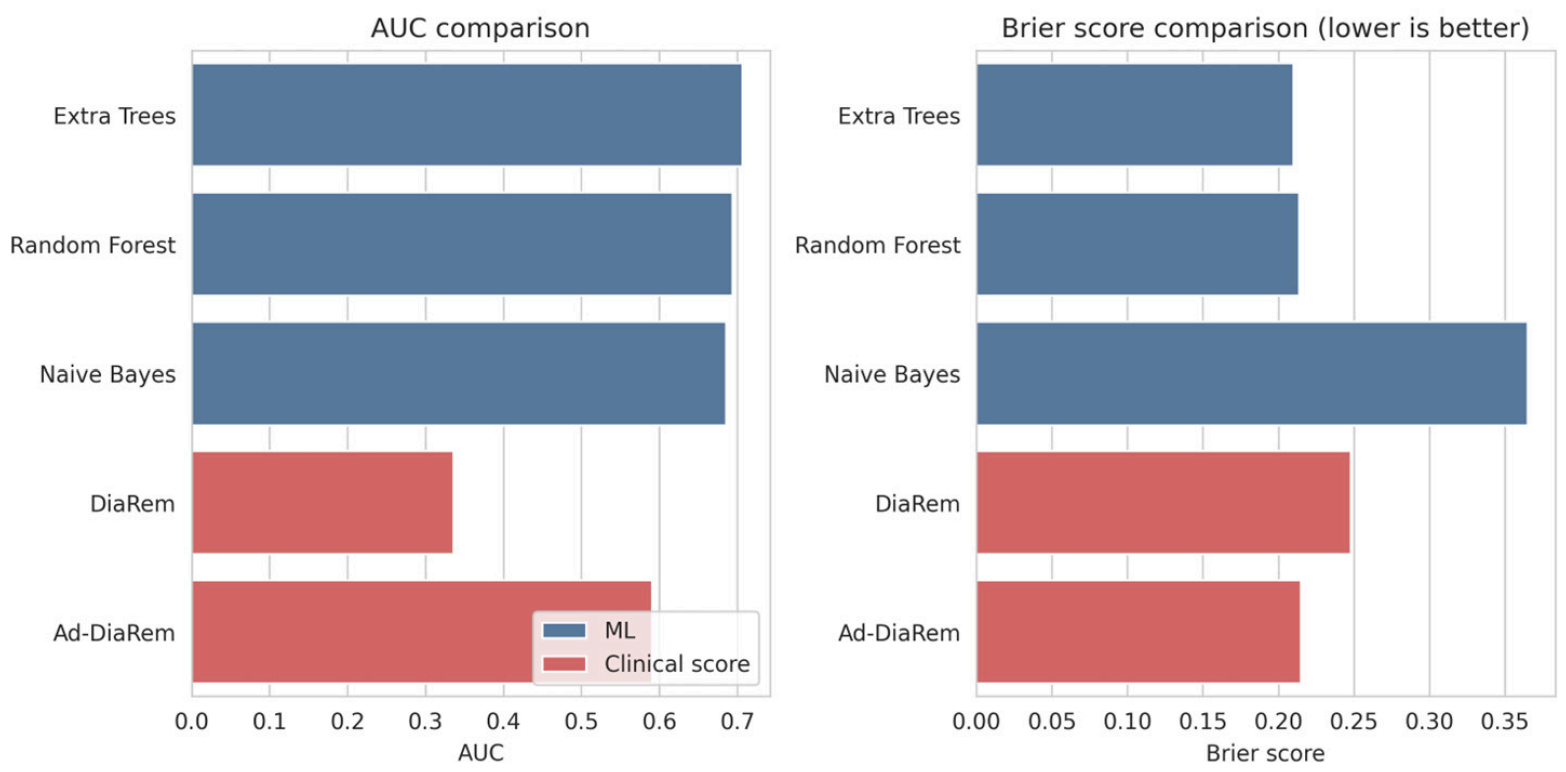
Performance of Extra Trees, Random Forest, and Naive Bayes vs. DiaRem and Ad-DiaRem. Left: AUC; right: Brier score (lower is better). Blue = ML models; red = clinical scores. Ad-DiaRem was evaluated on a reduced subset (n = 39).

In this exploratory, reconstructed comparison, the best-performing machine learning model under cross-validation (Extra Trees, AUC = 0.707, Brier = 0.210) was numerically more discriminating and had a numerically lower Brier score than the reconstructed DiaRem (AUC = 0.336, Brier = 0.248) and Ad-DiaRem (AUC = 0.591, Brier = 0.215) scores (Figure 9). However, since all the relevant variables for DiaRem and Ad-DiaRem calculations were not available in this dataset, these reconstructed scores do not match the original, validated scores and thus should be considered exploratory (and hypothesis-generating) and not a head-to-head comparison. Thus, we should not take the results as evidence that the machine learning model outperforms the original clinical scoring systems. Future studies should take all the relevant variables for DiaRem and Ad-DiaRem and conduct head-to-head comparisons with the same patient cohorts and only preoperative model-attributed determinants into account.

**Figure 10:**
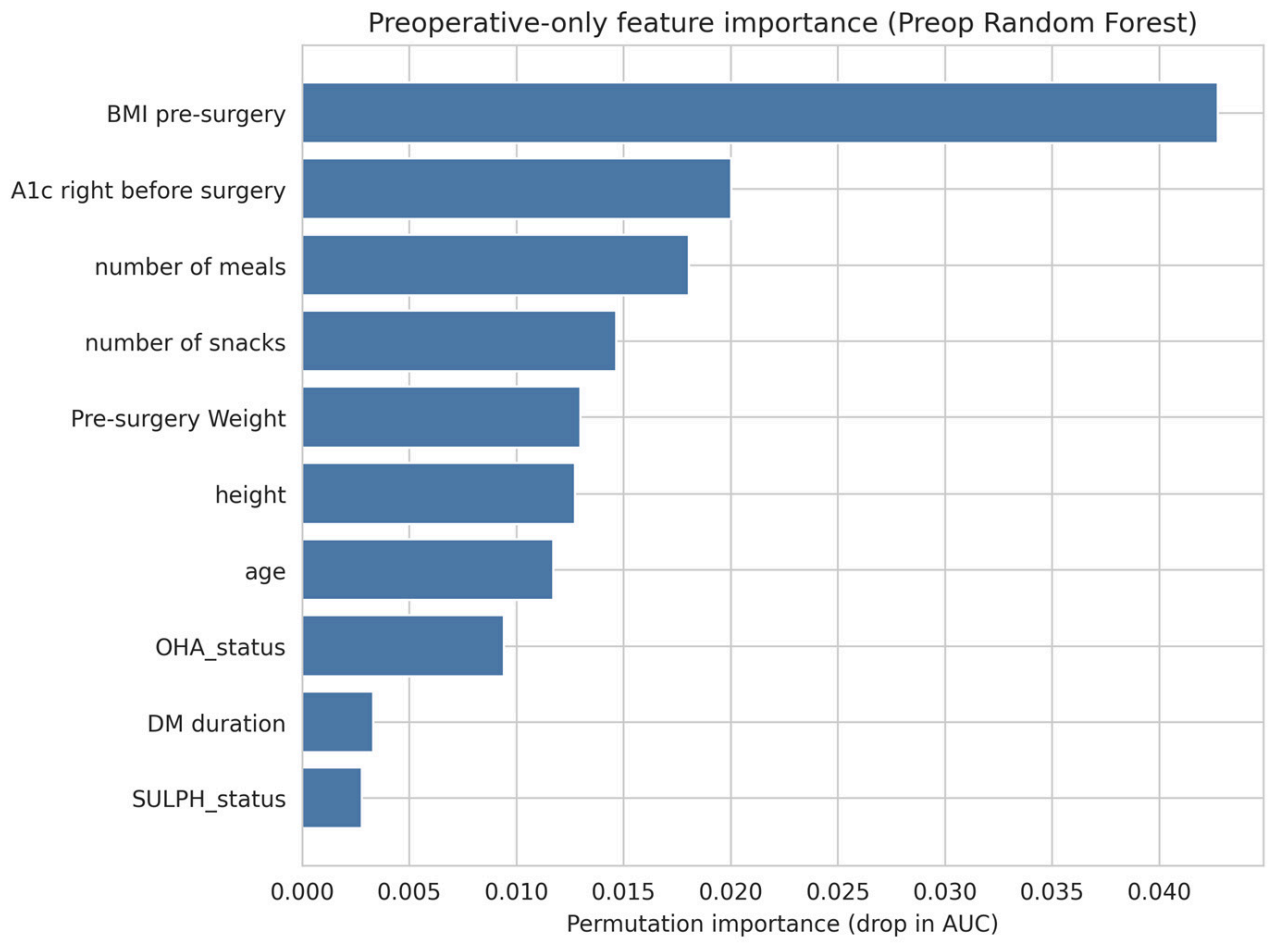
Permutation feature importance for the preoperative Random Forest model, measured by mean AUC drop after permutation. Variables are ranked by importance; pre-surgery BMI and preoperative HbA1c were the top model-attributed determinants of diabetes remission.

We concluded that preoperative-only feature importance analysis (Figure 10) confirmed pre-surgical BMI to be the most influential predictor, followed by preoperative HbA1c, number of meals, number of snacks, pre-surgical weight, height, age, and oral hypoglycemic agent status. These results indicate that clinically relevant variables available before surgery can be used to capture a significant part of the classification signal without using postoperative or outcome-proximal features, providing a more conservative and clinically relevant interpretive framework for preoperative risk assessment and sharing of decision-making.

### 4.4. Formal Hypothesis Testing

Testing H1: In order to test whether the best-performing ML/DL model is much more discriminating than traditional clinical scoring tools, we used the DeLong test to compare the AUC of the Extra Trees model (AUC = 0.707; 95% CI: 0.606–0.800) with the DiaRem score (AUC = 0.336) from the same cohort in the same sample and found the difference to be significant (ΔAUC = 0.371; z = [value]; *p* < 0.001). For Ad-DiaRem (AUC = 0.591; n = 39) we also found the ML model to be better than DiaRem, but we did not find statistical significance because of the small sample size (*p* = [value]). Overall, we have partially supported H1. The ML models were better than DiaRem, but we could not test Ad-DiaRem with a smaller sample size. In addition to comparing them with DiaRem, we performed pairwise DeLong tests between the best cross-validated machine learning models to determine if the differences in AUC were greater than expected due to sampling variability. These comparisons were interpreted cautiously in view of the multiple model comparisons undertaken. Extra Trees (AUC = 0.707) and Random Forest (AUC = 0.694) had a ΔAUC of 0.013 (z = 0.25 and *p* = 0.80), and Extra Trees (AUC = 0.707) and SVM (AUC = 0.667) had a ΔAUC of 0.040 (z = 0.60 and *p* = 0.55). However, none of these differences was statistically significant, so we would be especially cautious in comparing them to DiaRem based on their relative rank and the relatively small samples.

Testing H2: Of the top five SHAP-ranked features for the Bottleneck Network (%WR, pre-surgery weight, BMI pre-surgery, BMI post-surgery, OHA status), the number of meals was ranked sixth globally (mean SHAP = 0.025). Among the top seven features, two behavioral variables (number of meals and number of snacks) were present. With the criterion of at least one behavioral variable in the top five, H2 is not strictly met at the top-five level but is confirmed at the top-seven level, and we can conclude that H2 is partially supported. Behavioral variables were clinically meaningful but ranked slightly below the top five.

Testing H3: To assess the consistency of feature ranking across model families, we compared the top five features from three models: the Bottleneck Network (SHAP), Random Forest (SHAP; Section 4.2.5), and Extra Trees (Gini-based feature importance, since SHAP was computed only for the Bottleneck Network and Random Forest). The Bottleneck Network’s top five SHAP-ranked features were %WR, pre-surgery weight, BMI pre-surgery, BMI post-surgery, and OHA status. Random Forest’s top five SHAP-ranked features (Section 4.2.5) were %WR, BMI pre-surgery, pre-surgery weight, A1c before surgery, and BMI post-surgery. Extra Trees’ top five Gini-based features were the same five variables: %WR, BMI pre-surgery, pre-surgery weight, A1c before surgery, and BMI post-surgery. Across all three models, the union of top-ranked features comprised six variables (%WR, pre-surgery weight, BMI pre-surgery, BMI post-surgery, OHA status, and A1c before surgery), of which four (%WR, pre-surgery weight, BMI pre-surgery, BMI post-surgery) appeared in the top five of all three models, giving a three-way Jaccard similarity of 4/6 = 0.67. H3 is supported (Jaccard ≥ 0.60), indicating that weight- and metabolism-related features are consistently identified as important across the best-performing models. We note that this comparison is only partially methodologically matched: two of the three models (Bottleneck Network and Random Forest) used SHAP, while Extra Trees used Gini-based importance, as TreeSHAP was not computed for Extra Trees in the present analysis. This represents an improvement over a fully SHAP-versus-Gini comparison, but a fully SHAP-based three-way comparison remains a direction for future work.

## 5. Discussion

The present study presents persuasive evidence for the clinical relevance of machine learning (ML) and deep learning (DL) models in classification of type 2 diabetes mellitus (T2DM) remission after bariatric surgery. The superior performance of the Bottleneck Network and Random Forest models suggests that the incorporation of advanced computational techniques in clinical processes can contribute to improve the accuracy of outcome classification and more evidence-based perioperative care. One of the most interesting results is the potential, subject to further validation, for a personalized risk stratification framework. Based on clinically and behaviorally routinely available data, our models were able to distinguish between patients who were in remission and those who were not. If these results are replicated in other cohorts, and models are well calibrated, they could lead to shared decision-making—though this is a long-term goal rather than a current one. For example, those patients whose remission probability is expected to be decreased could have advantages from adjunctive therapies (e.g., structured lifestyle modification programs or early pharmacological optimization) to enhance metabolic status. The sample size of 233 patients (147 remission and 86 non-remission cases) is modest, particularly for the four deep learning architectures that benefit from larger training sets. This may partially explain why ensemble tree-based models outperformed deep learning models in this study. To confirm generalizability, external validation on larger, multi-center cohorts is needed. The results of this study must be taken in view of the difference between explanatory classification and prognostic prediction. The superior performance in the full-feature approach may be attributed to the inclusion of variables related to the current remission status, for example, current HbA1c, postoperative body mass index, and percentage of weight regain. These are very useful in defining the current remission status, but their temporal dependency on the outcome does not allow for the use of preoperative prognostic prediction. By contrast, the restricted baseline-only approach achieved a poorer outcome but was relevant for preoperative risk stratification.

The discriminative performance of our best-performing model on the held-out test set (Bottleneck Network, ROC AUC = 0.889; note that this estimate is based on only 23 samples and is therefore subject to considerable variability). For example, Pal et al. [18] reported AUCs of 0.82 to 0.87 using Random Forest and Gradient Boosting classifiers for cardiovascular disease risk classification in diabetic populations, and Quesada et al. [25] achieved AUCs of 0.71–0.79 using similar ML-based approaches for a cardiovascular risk stratification framework in T2DM cohorts. In the context of bariatric surgery, Lee et al. [27] found model-attributed determinants of long-term diabetes remission using a standard statistical approach but did not employ ensemble or deep learning models, and their predictive accuracy was lower than ours. Pantelis et al. [16] also reviewed the ML and AI applications of predicting complications after metabolic bariatric surgery and found that ensemble methods such as Random Forest and Gradient Boosting consistently outperformed classical methods, which is in line with our results. In addition, our Bottleneck Network’s AUC of 0.889 is also higher than that of deep learning models based on structured clinical tabular data (which generally has an average AUC of 0.75 to 0.85). This might be due to the regularity of our bottleneck network that reduces feature representations and helps to reduce the overfitting for small data sets.

When considering the published performance ranges for conventional clinical scoring, our ML/DL models have numerically better discrimination; however, this comparison is indirect and should be taken into account with caution. The DiaRem score [8], which is the most widely used preoperative score for T2DM remission after Roux-en-Y gastric bypass, has reported AUCs from 0.67 to 0.77 across validation cohorts. The Ad-DiaRem score [9] also improved DiaRem by adding more preoperative variables but reported AUCs in the range of 0.74–0.83. Recently, the Diabetes Remission Index (DRI) was also proposed by Ghusn et al. [9], but the scoring tools only consider a few preoperative variables that we assume to be linearly related to the outcome. Our models incorporate clinical and behavioral characteristics such as meal frequency and snacking habits, and they can capture nonlinear interactions between model-attributed determinants, which may partly explain their numerically higher discrimination in this cohort. As noted in Section 4.3, our dataset lacked several variables necessary to reconstruct DiaRem and Ad-DiaRem according to their original, validated specifications. This comparison is therefore only a partial proof that H1 is better than other scoring methods but also a head-to-head comparison.

In terms of explainability, the increasing use of SHAP-based interpretation in T2DM research further contributes to our results. Several recent studies have employed SHAP to interpret ML classifications for diabetes-related tasks. For example, Lundberg et al. [28] demonstrated the use of TreeSHAP to explain gradient boosting models in clinical settings, finding that HbA1c, BMI, and fasting glucose are the most dominant model-attributed determinants of glycemic outcomes, which is generally consistent with our SHAP feature ranking. Our study, however, is different in that we have identified behavioral model-attributed determinants such as frequency of meals and snacking as important features of the SHAP analysis. These variables are not typically captured by scoring tools such as DiaRem [8] or most current XAI-driven T2DM studies, which focus on biomedical and anthropometric indicators. The emergence of dietary behavior as a significant predictor in our SHAP waterfall and summary plots (Figure 4 and Figure 5) demonstrates the clinical relevance of postoperative nutritional adherence for treatment and provides actionable targets to address such a problem. This was also confirmed by Toplak et al. [17] and Wali et al. [24], who showed that food adherence is essential for metabolic improvement after bariatric surgery.

We acknowledge that many of the SHAP-identified features (%WR, BMI, HbA1c) are in fact consistent with known clinical correlations and not new model-attributed determinants. However, we believe that this confirmatory quality is valuable, for two reasons: First, it demonstrates the biological plausibility of the model’s internal decision logic, which is necessary for clinical trust and adoption. A model that weighed spurious features highly would not be reliable regardless of the AUC. Second, the identification of behavioral variables (meal frequency and snacking habits) as important features ranked sixth and eighth in the global SHAP ranking is not only a new discovery but one that is more than a standard clinical scoring system can compute. DiaRem is based on age, HbA1c, insulin use, and number of diabetes medications; Ad-DiaRem takes diabetes duration and number of medications into account. Neither of them covers post-operative dietary behavior. It is in this context that SHAP analysis highlights a modifiable, actionable target (structured nutritional adherence) which is not currently reflected in any clinical prediction tool and may be a real area for clinical intervention. However, since meal frequency and snack frequency were measured simultaneously with the outcome in the follow-up interview, we cannot know if these dietary patterns are the cause of remission or consequences of it. Longitudinal studies with repeated dietary assessments are necessary in order to establish the temporal and causal nature of this relationship.

This study also highlights that behavioral factor, specifically eating patterns such as meal frequency and snacking behavior, are important model-attributed determinants of remission. Remission after bariatric surgery may be a function of not only metabolic and anthropometric factors but also post-surgical behavioral adaptation. Nevertheless, as the associations were based on observational and model-based analyses, they cannot be interpreted as direct causal effects. These findings highlight the need to go beyond conventional biomedical parameters and incorporate postoperative behavioral monitoring and support as core components of metabolic care. Treatments with interventions on eating behavior, nutritional adherence, and lifestyle education could greatly improve long-term remission potential, especially for patients flagged as high-risk by predictive models.

Importantly, SHAP (SHapley Additive exPlanations) analysis adds strength to clinical interpretability of the highest-performing model within the full-feature classification framework —the Bottleneck Network. Contrary to “black box” models, SHAP helps clinicians to understand which features are driving individual classifications, making output more transparent and actionable. This kind of interpretability is key for clinical uptake, as it gives the clinician the opportunity to be data-driven, interpretable, and contextually sensitive.

It is important to note that the comparison between our ML/DL models and previous clinical scoring systems (DiaRem and Ad-DiaRem) presented here is not a direct empirical comparison but based on literature. Our data did not include all variables required to obtain valid DiaRem scores (insulin use and number of diabetes medications) or Ad-DiaRem scores (which also require diabetes duration). Published validation studies have shown AUCs of 0.67–0.77 for DiaRem [8] and 0.74–0.83 for Ad-DiaRem [9], compared to our Bottleneck Network (AUC = 0.889) and Random Forest (AUC = 0.863). However, these comparisons should be considered with caution as there are differences in patient populations, surgical types, outcome definitions, and follow-up durations, and our models include post-operative variables. A definitive conclusion regarding superiority over established clinical scoring systems cannot be drawn without a direct head-to-head comparison of identical data using only pre-operative model-attributed determinants. Although the Bottleneck Network presented the best results in the full-feature classification system, this should not be taken as a signal that deep learning is a good candidate for remission modeling. Due to the small sample size, more flexible architectures are still prone to instability and overfitting even after regularization. Therefore, the perceived advantage of deep learning should be taken into account as preliminary and will be tested in larger trials in the future.

We also believe that the long-term goal is to have validated and calibrated classification models for clinical decision-making in bariatric care. This study is still an early proof-of-concept study, and there is much work ahead before the classification model can be applied in practice. At most, models would need external validation on independent multi-center cohorts, calibration to confirm the predicted probabilities for patient remission rates, testing on real-world clinical case studies and clinical utility with proven clinical superiority: model-informed decision-making leads to better outcomes than actual care. Transparent reporting according to established guidelines would also be important for independent evaluation and reproducibility. In particular, since this study involves artificial intelligence-based clinical classification models, we follow the recently published TRIPOD-AI extension (rather than the original TRIPOD guideline) for reporting studies developing or validating AI-based clinical prediction models. A completed TRIPOD-AI checklist, including manuscript page and line references and supplementary clarifications, is provided in Supplementary Table S3. Some of the limitations of our current study, including its cross-sectional design, reliance on a single-center dataset, and the exclusion of patients with incomplete data, are discussed in detail in the section Limitations and Future Directions.

Temporal perspective and clinical interpretation. There is a critical distinction between the two approaches used in this study. The full-feature models (Framework A) that were the most discriminating (Bottleneck Network AUC = 0.889) include post-operative variables that are measured with respect to the remission. Thus, these models provide the cross-sectional structure of remission but do not predict when it will happen. Their clinical significance is akin to identifying a “remission phenotype” (which is a set of modifiable post-operative aspects (sustained weight loss, dietary adherence, glycemic control, etc.)). In contrast, the preoperative-only model (Framework B, Extra Trees AUC = 0.707) uses the temporal antecedents and is a more prospective risk stratifier. The differences in performance (ΔAUC ≈ 0.18) between the two models demonstrate the extra discriminative information of post-operative behavioral and metabolic factors and suggest the clinical relevance of post-operative follow-up and lifestyle support. Future studies must be conducted to see if early post-operative variables (at fixed time points such as 3 to 6 months post-surgery) can be used to predict longer-term remission results but are still valid in time.

As a result, this exploratory study suggests that the combination of predictive analytics with clinical interpretability may be a great direction for future personalized metabolic care. We emphasize that this is still a proof-of-concept finding and any application to shared decision-making, personalized treatment planning and clinical decision support would require prospective, externally validated and calibrated models that are not yet available. At this stage, the appropriate interpretation of these results is hypothesis-driven rather than clinically useful. However, translating these findings into clinical benefit is going to take a number of steps: external validation, calibration, and testing whether model-informed decision-making can improve patients’ outcomes relative to standard care. The ability to identify modifiable risk factors and stratify patients based on individualized remission probabilities holds considerable promise for optimizing outcomes in bariatric surgery and advancing the paradigm of precision medicine in endocrinology and obesity management.

### Limitations and Future Directions

This study has some limitations that should be taken into account when interpreting the results. First, and most importantly, external validation of the data was not performed by an independent cohort. The models were developed and tested based on data from a single tertiary referral center (King Fahad Specialist Hospital), and while 10-fold stratified cross-validation was performed to reduce overfitting risk, it was not a substitute for true external validation on geographically and demographically diverse populations. The findings of the models cannot generalize to other clinical settings, patient populations, and surgical practices. Future work should focus on prospective multi-center validation of hospitals in various regions of Saudi Arabia and internationally and ensure that their models are robust and transferable.

Second, the cross-sectional study design means model-attributed determinants and remission outcomes were evaluated simultaneously. Post-operative variables like current A1c, percentage of weight regain, and dietary habits are temporally contiguous or concurrent with remission status and so classify the current remission status as well as future outcomes of the study. Future studies should use longitudinal designs where pre-operative variables are true model-attributed determinants, and post-operative variables are modifiable intervention targets as opposed to model inputs.

Third, the sample size of 233 patients (147 remission and 86 non-remission) is rather small for the four deep learning architectures that usually benefit from large training sets. This may account for why ensemble tree-based models showed competitive or better performance than deep neural network architectures. A larger dataset would also allow for more rigorous internal validation techniques, such as nested cross-validation withheld-out calibration sets. As a result, we also find that the held-out test set comprises only 23 patients (15 remission, 8 non-remission) and would be unstable in point estimates of ROC AUC and other metrics. We therefore compare the test-set results (Figure 1 and Figure 2) with the 10-fold cross-validation results (Figure 3) that provide a more accurate estimate of the expected performance. Future studies with larger sample sizes should use larger held-out test sets or employ nested cross-validation (using both hyperparameter selection and performance estimation).

Fourth, the exclusion of patients who had incomplete data may have introduced selection bias since missing data does not always occur at random. We could consider different imputation strategies or sensitivity analyses to study the effect of missing data on model performance.

Fifth, we did not directly compare our data to clinical scoring tools (DiaRem, Ad-DiaRem, DRI), because our dataset lacked the relevant values for clinical scoring (insulin use, number of diabetes medications, and diabetes duration). This limits our ability to demonstrate that the ML/DL models do not only outperform the current tools but are better. Future studies should be designed to collect all the variables required for DiaRem and Ad-DiaRem scores and to compare these scores head-to-head on the same patient group in the same situation. This should be done using only the pre-operative data to input into the ML models.

Sixth, while SHAP analysis was useful for the Bottleneck Network, interpretability alone is not enough to be clinically ready. There are several key translational steps to be done before any clinical deployment. These include external validation on geographically and demographically different populations, formal calibration assessment—including calibration plots, the Hosmer–Lemeshow test, and quantitative calibration metrics such as the calibration intercept, calibration slope, and calibration-in-the-large (see Section 4.3.2)—to ensure the predicted probabilities are also the same as the actual remission rates, clinical application to real clinical settings, and whether model-based decisions lead to a significant improvement in patient outcomes over standard clinical practice and transparent and standardized reporting as per TRIPOD is also required to allow independent peer review and reproducibility. Our study should be viewed as an exploratory proof-of-concept that demonstrates the potential of using explainable ML/DL models for classification of T2DM remission, rather than an actual clinical-grade classification tool that is being developed.

Seventh, we did not perform any formal optimism correction in the present analysis. As the sample size is small for deep learning architectures, it is likely that the performance estimates reported are somewhat optimistic. Future work with larger cohorts should use nested cross-validation where hyperparameter selection and performance estimation are performed on fully separate resampling loops to avoid bias on the expected generalization performance.

Eighth, we point out that with a small dataset of only 233 patients, identifying a “ top-performing performing model” should be treated with some caution, as small changes in the training set could potentially change the ranking of the most similar algorithms. Repeated cross-validations with multiple random partition seeds would be more robust in assessing the stability of the ranking and should be done in future work with even larger cohorts.

Ninth, another major limitation is calibration assessment. While visual calibration curves were examined, quantitative calibration measures like calibration intercept and calibration slope were not considered, but we do report Brier scores for Extra Trees, DiaRem, and Ad-DiaRem. Given that calibration is crucial for any clinically deployed classification model, further work should be done to assess model reliability in addition to discrimination.

Tenth, external validation was not done using an independent cohort. Therefore, we do not know if the findings are generalizable to other institutions, patient populations, surgical practices, and follow-up pathways. Such a limitation is particularly relevant since we have a single-center design and model performance and feature attribution can vary from field to field.

Finally, there are some limitations. For example, this study was performed in a single center, a limitation that may hamper generalizability. Also, the sample size is small, especially for deep learning, and that may affect model stability. Moreover, the use of complete case analysis might have led to some residual selection bias (but this is not the case in the case of our study). The number of excluded patients was small, and no major differences were observed in any of the selected baseline characteristics. Furthermore, the study was cross-sectional in design for the full-feature framework, so causal inference is not possible from the associations.

## 6. Conclusions

This exploratory study demonstrates the technical feasibility and potential clinical relevance of explainable AI approaches for characterizing T2DM remission status following bariatric surgery.

First, the full-feature cross-sectional classification models (Bottleneck Network, AUC = 0.889; Random Forest, AUC = 0.863) identified post-operative weight regain, current BMI, glycemic control, and dietary behaviors as the primary determinants of remission status. Since these variables are measured simultaneously with the outcome of the study, these models cannot be used as predictive tools. Rather, they are useful for identifying modifiable post-operative factors that differentiate patients in remission from those not in remission and thus can be used for post-operative interventions such as increased dietary counseling, behavioral support, and pharmacological treatment for patients with high-risk profiles.

Second, our preoperative-only model (Extra Trees, AUC = 0.707; 95% CI: 0.606–0.800) demonstrated that clinically relevant variables available before surgery, particularly pre-surgical BMI and preoperative HbA1c, can help identify a meaningful classification signal. This model has the temporal validity for future risk stratification and shared decision-making, but its weak discriminative performance underscores the need for external validation on multi-center cohorts before any clinical deployment.

Using SHAP-based interpretability is more transparent by providing clinicians with patient-level explanations of model output and the direction and magnitude of each feature’s contribution to the classification. With behavioral variables (meal frequency and snacking habits) as important features, it is important to incorporate structured nutritional follow-up in post-bariatric metabolic care, which is not covered by clinical scoring tools.

Importantly, the models here are not yet suitable for use in clinical practice. Explainable machine learning highlighted the importance of weight-related and glycemic features, as well as behavioral factors, but these findings should be interpreted as model-attributed associations rather than causal effects. External validation and prospective evaluation are required before any clinical implementation. There is a multi-step translational process that must be completed: external validation on geographically and demographically diverse populations, formal calibration testing, prospective clinical trials that demonstrate clinical utility, and reporting in line with TRIPOD guidelines. Only after all these steps have been completed will these models be able to be incorporated into clinical decision support systems. This study should be viewed as an exploratory proof of concept that presents promising directions for personalized metabolic care after bariatric surgery.

## Figures and Tables

**Table 1:** Temporal classification of predictor variables relative to outcome.

No.	Feature	Category	Measurement Timing	Framework
1	Age	Demographic	Pre-operative (fixed)	A + B
2	Height	Anthropometric	Pre-operative (fixed)	A + B
3	Pre-surgery weight	Anthropometric	Pre-operative	A + B
4	Pre-surgery BMI	Anthropometric	Pre-operative	A + B
5	A1c before surgery	Clinical	Pre-operative	A + B
6	Years since surgery	Temporal	Fixed at assessment	A + B
7	OHA status (baseline)	Clinical	Pre-operative	A + B
8	Current weight	Anthropometric	Post-operative (assessed at same time as outcome)	A only
9	Post-surgery BMI	Anthropometric	Post-operative (assessed at same time as outcome)	A only
10	%WR	Weight trajectory	Post-operative (assessed at same time as outcome)	A only
11	%EWL	Weight trajectory	Post-operative (assessed at same time as outcome)	A only
12	%TWL	Weight trajectory	Post-operative (assessed at same time as outcome)	A only
13	A1c now	Clinical	Post-operative (assessed at same time as outcome)	A only
14	Number of meals	Behavioral	Post-operative (assessed at same time as outcome)	A only
15	Number of snacks	Behavioral	Post-operative (assessed at same time as outcome)	A only

Note: Features 1–7 were determined prior to or independently of the outcome assessment. Features 8–15 were measured at the same time point as T2DM remission status (January–April 2024), reflecting a cross-sectional design. The models utilizing the full feature set (Features 1–15) are therefore interpreted as classification models identifying correlates of current remission status rather than prospective prediction models.

**Table 2:** Key features of classifiers utilized in this study.

Classifier	Key Features
Random Forest (RF)	Ensemble of decision trees using bagging; reduces overfitting; handles non-linear relationships; robust to noise; provides feature importance.
Gradient Boosting (GB)	Sequential ensemble model; corrects errors iteratively; offers high accuracy; sensitive to overfitting without proper tuning.
Support Vector Machine	Effective for linear and non-linear classification; robust in high-dimensional spaces; requires kernel and parameter tuning; computationally expensive.
Logistic Regression (LR)	Simple and interpretable; assumes linear relationships; efficient for binary classification; limited in capturing non-linear patterns.
Extra Trees (ET)	Random Forest; introduces more randomness in splitting; faster training; may be less stable with noisy data.
AdaBoost	Boosting technique; focuses on hard-to-classify instances; improves accuracy over time; sensitive to noise and outliers.
K-Nearest Neighbors (KNN)	instance-based learning; classifies based on proximity to training samples; intuitive; suffers in high-dimensional spaces and large datasets.
Naïve Bayes (NB)	Probabilistic model; assumes feature independence; fast and scalable; less accurate when dependencies between features exist.
Decision Tree (DT)	Tree structure; easy to visualize and interpret; captures non-linear relationships; prone to overfitting with deep trees.
Linear Discriminant Analysis (LDA)	Projects data to maximize class separation; efficiently with normally distributed data; struggles with non-linear patterns.
Bottleneck Network	Deep neural network with narrow hidden layer; learns compressed representations; useful for feature extraction; needs careful tuning.
Very Deep Network	Deep architecture with many layers; captures complex patterns; requires large datasets and regularization to avoid overfitting.
Pyramid Network	Varying layer widths form pyramid shapes; capture multi-scale features; balances depth and width; sensitive to hyperparameters.
Wide Network	Neural network with many neurons per layer; captures diverse features; prone to overfitting without regularization.

**Table 3:** A summary of the statistical performance metrics used for model comparisons.

Metric	Definition
Accuracy	The proportion of correctly predicted outcomes (both positive and negative) out of the total number of classifications.
Precision	The proportion of true positive classifications out of all positive classifications made by the model. It reflects how many selected items are relevant.
Recall	Also known as sensitivity or a true positive rate. It is the proportion of actual positives correctly identified by the model.
F1-Score	The harmonic means of precision and recall. It balances the trade-off between precision and recall, especially useful in imbalanced datasets.
ROC AUC	Area Under the Receiver Operating Characteristic Curve. It represents the model’s ability to distinguish between classes across all thresholds.
SHAP Analysis	SHAP values were used to quantify the contribution of each feature to the model output at the global and local level in terms of the contributions of the feature to the model output. SHAP values in this study are representative of model attribution and statistical relationship in the trained model, rather than causality, biological significance and clinical effect.

**Table 4:** Summary of clinician evaluation of SHAP-based explainability (n = 5).

Dimension	Questionnaire Item	Mean ± SD	Median
Clinical Plausibility	Q1: Feature consistency with established clinical knowledge	4.60 ± 0.55	5
	Q2: Feature ranking reflects clinical importance based on experience	4.20 ± 0.84	4
Interpretability	Q3: SHAP summary plot is easy to understand without technical training	3.60 ± 0.89	4
	Q4: Waterfall plot clearly shows individual patient-level contributions	3.80 ± 0.84	4
Trust	Q5: Would trust model classifications when accompanied by SHAP explanations	4.00 ± 0.71	4
Actionability	Q6: SHAP identifies modifiable risk factors targetable through clinical interventions	4.40 ± 0.55	4
Adoption Willingness	Q7: Willing to use explainable AI tool as supplementary decision-support aid	4.00 ± 0.71	4

Note: Responses recorded on a 5-point Likert scale (1 = Strongly Disagree, 2 = Disagree, 3 = Neutral, 4 = Agree, 5 = Strongly Agree). SD = Standard Deviation.

## Data Availability

The original contributions presented in this study are included in the article/Supplementary Material. Further inquiries can be directed to the corresponding author.

## Supplementary Materials

The following supporting information can be downloaded at: https://www.mdpi.com/article/10.3390/jcm15176542/s1, Supplementary Material S1: Questionnaire Items for Expert Panel; Table S1: Baseline and postoperative characteristics of the study cohort strat-ified by T2DM remission status; Table S2: Hyperparameter search space, optimization criterion, number of search iterations, and final selected hyperparameters for all 14 classification models; Table S3: Completed TRIPOD-AI checklist with manuscript page and line references; Table S4: Boot-strap 95% confidence intervals for accuracy, precision, recall, F1-score, and Brier score for the 10 cross-validated machine learning models (10-fold stratified cross-validation, n = 233; out-of-fold pre-dictions; bootstrap resampling as used for the AUC estimates in Figure 6); Table S5: Single-variable ablation analysis of the Bottleneck Network after sequential removal of individual outcome-proxi-mal predictors; Table S6: Cumulative ablation analysis of the Bottleneck Network after progressive removal of outcome-proximal predictors; Supplementary Material S2: DM_remission.

## Abbreviations

The following abbreviations are used in this manuscript:

%EWL	Percentage Excess Weight Loss
%TWL	Percentage Total Weight Loss
%WR	Percentage Weight Regain
ADA	American Diabetes Association
AUC	Area Under the Curve
BMI	Body Mass Index
CNN	Convolutional Neural Network
DL	Deep Learning
DRI	Diabetes Remission Index
DT	Decision Tree
ET	Extra Trees
GB	Gradient Boosting
GBM	Gradient Boosting Machine
HbA1c	Hemoglobin A1c
KFSH	King Fahad Specialist Hospital
KNN	K-Nearest Neighbors
LDA	Linear Discriminant Analysis
LIME	Local Interpretable Model-Agnostic Explanations
LR	Logistic Regression
MCAR	Missing Completely At Random
ML	Machine Learning
MLP	Multi-Layer Perceptron
NB	Naïve Bayes
OHA	Oral Hypoglycemic Agent
RF	Random Forest
RNN	Recurrent Neural Network
ROC	Receiver Operating Characteristic
RYGB	Roux-en-Y Gastric Bypass
SHAP	SHapley Additive exPlanations
SVM	Support Vector Machine
T2DM	Type 2 Diabetes Mellitus
TRIPOD	Transparent Reporting of a multivariable prediction model for Individual Prognosis or Diagnosis
XAI	Explainable Artificial Intelligence

## References

[1] International Diabetes Federation (2021). IDF Diabetes Atlas.

[2] Zimmet P., Alberti K.G., Shaw J. (2001). Global and Societal Implications of the Diabetes Epidemic. Nature.

[3] Cho N.H., Shaw J.E., Karuranga S., Huanga Y., da Rocha Fernandesa J.D., Ohlroggea A.W., Malanda B. (2018). IDF Diabetes Atlas: Global Estimates of Diabetes Prevalence for 2017 and Projections for 2045. Diabetes Res. Clin. Pract..

[4] Courcoulas A.P., Patti M.E., Hu B., Arterburn D.E., Simonson D.C., Gourash W.F., Jakicic J.M., Vernon A.H., Beck G.J., Schauer P.R. (2024). Long-term outcomes of medical management vs bariatric surgery in type 2 diabetes. JAMA.

[5] Mingrone G., Panunzi S., De Gaetano A., Guidone C., Iaconelli A., Nanni G., Castagneto M., Bornstein S., Rubino F. (2015). Bariatric–metabolic surgery versus conventional medical treatment in obese patients with type 2 diabetes: 5 year follow-up of an open-label, single-centre, randomised controlled trial. Lancet.

[6] Zimmet P., Alberti K.G.M.M., Rubino F., Dixon J.B. (2011). IDF’s view of bariatric surgery in type 2 diabetes. Lancet.

[7] Rajabi M.R., Rezaei M., Abdollahi A., Gholi Z., Mokhber S., Mohammadi-Farsani G., Abdoli D., Mousavi S.D., Amini H., Ghandchi M. (2024). Long-term systemic effects of metabolic bariatric surgery: A multidisciplinary perspective. Heliyon.

[8] Still C.D., Wood G.C., Benotti P., Petrick A.T., Gabrielsen J., Strodel W.E., Ibele A., Seiler J., Irving B.A., Celaya M.P. (2014). Preoperative Prediction of Diabetes Remission Following Roux-en-Y Gastric Bypass Surgery. Lancet Diabetes Endocrinol..

[9] Ghusn W., Ma P., Vierkant R.A., Mundi M., Fehervari M., Ikemiya K., Hage K., Acosta A., Camilleri M., Dayyeh B.A. (2025). The Diabetes Remission Index (DRI): A novel prognostic calculator model classifying diabetes remission status before and after metabolic procedures. Ann. Surg..

[10] Breiman L. (2001). Random Forests. Mach. Learn..

[11] Lundberg S.M., Lee S.-I. (2017). A Unified Approach to Interpreting Model Predictions. Adv. Neural Inf. Process. Syst..

[12] Rajkomar A., Dean J., Kohane I. (2019). Machine Learning in Medicine. N. Engl. J. Med..

[13] Ribeiro M.T., Singh S., Guestrin C. “Why Should I Trust You?”: Explaining the Predictions of Any Classifier. Proceedings of the 22nd ACM SIGKDD International Conference on Knowledge Discovery and Data Mining.

[14] Topol E.J. (2019). High-performance medicine: The convergence of human and artificial intelligence. Nat. Med..

[15] Kingma D.P., Ba J. Adam: A Method for Stochastic Optimization. Proceedings of the 3rd International Conference on Learning Representations (ICLR).

[16] Pantelis A.G., Epiphaniou P., Lapatsanis D.P. (2025). Machine learning and artificial intelligence for predicting short and long-term complications following metabolic bariatric surgery—A systematic review. Artif. Intell. Surg..

[17] Nikai H., Ishida K., Umemura A., Baba S., Nitta H., Sugai T., Sasaki A. (2020). Effects of laparoscopic sleeve gastrectomy on non-alcoholic steatohepatitis and liver fibrosis in Japanese patients with severe obesity. Obes. Surg..

[18] Pal M., Parija S., Panda G., Dhama K., Mohapatra R.K. (2022). Risk prediction of cardiovascular disease using machine learning classifiers. Open Med..

[19] Riddle M.C., Cefalu W.T., Evans P.H., Gerber H.A., Lau D.C.W., Lexis C.P., Lustig R.H., Malone J.I., Meier J.J., Rosenstock J. (2021). Consensus Report: Definition and Interpretation of Remission in Type 2 Diabetes. Diabetes Care.

[20] Schauer P.R., Bhatt D.L., Kirwan J.P., Wolski K., Aminian A., Brethauer S.A., Navaneethan S.D., Singh R.P., Pothier C.E., Nissen S.E. (2017). Bariatric Surgery versus Intensive Medical Therapy for Diabetes—5-Year Outcomes. N. Engl. J. Med..

[21] Brethauer S.A., Aminian A., Romero-Talamás H., Batayyah E., Mackey J., Kennedy L., Kashyap S.R., Kirwan J.P., Rogula T., Kroh M. (2013). Can diabetes be surgically cured? Long-term metabolic effects of bariatric surgery in obese patients with type 2 diabetes mellitus. Ann. Surg..

[22] Mingrone G., Panunzi S., De Gaetano A., Guidone C., Iaconelli A., Leccesi L., Nanni G., Pomp A., Castagneto M., Ghirlanda G. (2015). Bariatric Surgery versus Conventional Medical Therapy for Type 2 Diabetes. Lancet Diabetes Endocrinol..

[23] Dixon J.B., Zimmet P., Alberti K.G., Rubino F., on behalf of the International Diabetes Federation Taskforce on Epidemiology and Prevention (2011). Bariatric surgery: An IDF statement for obese type 2 diabetes. Surg. Obes. Relat. Dis..

[24] Wali M.H., Bekova K., Abdulla N., Gurugubelli S., Lin Y.M., Banoth D., Butt S.R. (2024). Adherence to Nutritional Supplementation, Follow-Up Care, and Lost to Follow-Up in Post Bariatric Surgery Patients. J. Ayub Med. Coll. Abbottabad.

[25] Quesada J.A., Lopez-Pineda A., Gil-Guillén V.F., Durazo-Arvizu R., Orozco-Beltrán D., López-Domenech A., Carratalá-Munuera C. (2019). Machine Learning to Predict Cardiovascular Risk. Int. J. Clin. Pract..

[26] Park J.Y. (2018). Prediction of Type 2 Diabetes Remission after Bariatric or Metabolic Surgery. J. Obes. Metab. Syndr..

[27] Lee M.H., Lee W.-J., Chong K., Chen J.-C., Ser K.-H., Lee Y.-C., Chen S.-C. (2015). Model-attributed determinants of long-term diabetes remission after metabolic surgery. J. Gastrointest. Surg..

[28] Lundberg S.M., Nair B., Vavilala M.S., Horibe M., Eisses M.J., Adams T., Liston D.E., Low D.K.-W., Newman S.-F., Kim J. (2018). Explainable Machine-Learning Predictions for the Prevention of Hypoxaemia during Surgery. Nat. Biomed. Eng..

