# Supplementary material for: Clinical and Behavioral Determinants of Type 2 Diabetes Remission After Bariatric Surgery: An Explainable Machine Learning Approach
Source: J Clin Med. 2026 Aug 24;15(17):6542. doi: 10.3390/jcm15176542 (PMC13566333; doi:10.3390/jcm15176542)
Supplement: Supplementary file 1 [file jcm-15-06542-s001.zip › S1 updated.pdf]

2. Questionnaire Items for Expert Panel

Clinician Evaluation Questionnaire: SHAP-Based Explainability Assessment

Instructions: We have been shown the SHAP summary plot, feature importance chart, and three individual patient waterfall plots generated by a deep learning model (Bottleneck Network) trained to predict T2DM remission after bariatric surgery. Please rate your agreement with each statement below on a scale from 1 (Strongly Disagree) to 5 (Strongly Agree).

Section A: Clinical Plausibility

Q1. The features identified as most important by the model (e.g., percentage weight regain, BMI, HbA1c) are consistent with established clinical knowledge about T2DM remission after bariatric surgery.

☐ 1 – Strongly Disagree ☐ 2 – Disagree ☐ 3 – Neutral ☐ 4 – Agree ☐ 5 – Strongly Agree

Q2. The relative ranking of features (e.g., weight regain ranked higher than age or height) reflects the clinical importance I would assign based on my experience.

☐ 1 – Strongly Disagree ☐ 2 – Disagree ☐ 3 – Neutral ☐ 4 – Agree ☐ 5 – Strongly Agree

Section B: Interpretability

Q3. The SHAP summary plot (beeswarm plot showing feature impact direction and magnitude) is easy to understand without extensive technical training.

☐ 1 – Strongly Disagree ☐ 2 – Disagree ☐ 3 – Neutral ☐ 4 – Agree ☐ 5 – Strongly Agree

Q4. The individual patient waterfall plot clearly shows which factors contributed to the predicted remission probability for that specific patient.

☐ 1 – Strongly Disagree ☐ 2 – Disagree ☐ 3 – Neutral ☐ 4 – Agree ☐ 5 – Strongly Agree

Section C: Trust and Reliability

Q5. I would trust a prediction from this model if it were presented alongside the SHAP explanation showing why that prediction was made.

☐ 1 – Strongly Disagree ☐ 2 – Disagree ☐ 3 – Neutral ☐ 4 – Agree ☐ 5 – Strongly Agree

Section D: Clinical Actionability

Q6. The SHAP-based explanations identify modifiable risk factors (e.g., dietary habits, weight regain) that I could target through clinical interventions for individual patients.

☐ 1 – Strongly Disagree ☐ 2 – Disagree ☐ 3 – Neutral ☐ 4 – Agree ☐ 5 – Strongly Agree

Section E: Willingness to Adopt

Q7. I would be willing to use this type of explainable AI tool as a supplementary decision-support aid in my clinical practice for bariatric surgery patients.

☐ 1 – Strongly Disagree ☐ 2 – Disagree ☐ 3 – Neutral ☐ 4 – Agree ☐ 5 – Strongly Agree

Section F: Open-Ended Questions

Q8. What aspects of the SHAP-based explanations did you find most useful or clinically relevant? Please explain.

____

Q9. What limitations, concerns, or improvements would you suggest before this tool could be used in clinical practice?

____

Clinician Demographics (for reporting purposes):

Specialty: ☐ Bariatric Surgery ☐ Endocrinology ☐ Other: ________

Years of clinical experience in bariatric/metabolic care: ________

Number of bariatric surgery patients managed annually (approximate): ________

Table S1. Baseline and postoperative characteristics of the study cohort stratified by T2DM remission status.

Variable	Remission (n = 42)	Non-Remission (n = 65)	Total (n = 107)
Demographics			
Age (years)	44.69 ± 11.82	43.97 ± 11.24	44.25 ± 11.43
Gender — Female, n (%)	26 (61.90%)	48 (73.85%)	74 (69.16%)
Gender — Male, n (%)	16 (38.10%)	17 (26.15%)	33 (30.84%)
Height (cm)	160.30 ± 9.68	160.18 ± 9.31	160.23 ± 9.42
Anthropometric and Weight Loss Metrics			
Pre-surgery weight (kg)	122.14 ± 23.76	113.04 ± 23.49	116.61 ± 23.89
Current weight (kg)	85.00 ± 16.53	79.52 ± 16.87	81.67 ± 16.85
BMI pre-surgery (kg/m^2)	47.23 ± 6.98	43.52 ± 8.32	44.98 ± 7.96
BMI post-surgery (kg/m^2)	33.14 ± 5.62	31.28 ± 5.74	32.01 ± 5.73
%EWL (Excess Weight Loss)	62.82 ± 23.48	70.18 ± 29.76	67.29 ± 27.56
EWL Success — Yes, n (%)	33 (78.57%)	43 (66.15%)	76 (71.03%)
%TWL (Total Weight Loss)	28.46 ± 11.53	27.24 ± 13.78	27.72 ± 12.92
%WR (Weight Regain)	21.12 ± 18.34	16.42 ± 22.58	18.26 ± 21.04
WR — Yes, n (%)	5 (11.90%)	3 (4.62%)	8 (7.48%)
Clinical and Glycaemic Parameters			
DM duration (years)[a]	12.55 ± 6.43	9.73 ± 5.81	10.71 ± 6.12
A1c before surgery (%)	7.01 ± 1.52	7.36 ± 1.82	7.22 ± 1.71
A1c after 6 months (%)[b]	5.68 ± 0.82	5.71 ± 0.93	5.70 ± 0.88

A1c current (%)[b]	5.86 ± 1.45	5.92 ± 1.56	5.90 ± 1.51
Metformin use — Yes, n (%)	12 (28.57%)	13 (20.00%)	25 (23.36%)
Sulphonylurea use — Yes, n (%)[c]	5 (11.90%)	5 (7.69%)	10 (9.35%)
Insulin use — Yes, n (%)[c]	3 (7.14%)	9 (13.85%)	12 (11.21%)
OHA use — Yes, n (%)[c]	13 (30.95%)	7 (10.77%)	20 (18.69%)
Lifestyle and Temporal Factors			
Number of meals/day	2.36 ± 0.79	2.08 ± 0.76	2.19 ± 0.78
Number of snacks/day	2.26 ± 0.89	2.55 ± 0.71	2.44 ± 0.80
Days since surgery	1330.67 ± 418.91	1314.26 ± 397.05	1320.70 ± 404.35
Years since surgery	3.81 ± 1.12	3.68 ± 1.05	3.73 ± 1.08

[a] DM duration was available for 33 patients only (11 remission, 22 non-remission); percentages are based on available data.

[b] A1c after 6 months and A1c current had missing values in several records; reported statistics are based on available data only.

[c] Sulphonylurea, Insulin, and OHA status had missing values in several records; counts reflect available data.

Continuous variables are reported as mean ± SD; categorical variables are reported as n (%).

Table S2. Hyperparameter search space, optimization criterion, number of search iterations, and final selected hyperparameters for all 14 classification models.

Model	Search Method	Optimization Criterion	CV / Validation Scheme	Search Iterations	Hyperparameter Search Space	Final Selected Hyperparameters
Logistic Regression (LR)	Grid Search	ROC AUC	5-fold CV	Exhaustive (all grid combinations)	C: [0.01, 0.1, 1, 10, 100]; penalty: [l1, l2]; solver: [liblinear, saga]	C = 1; penalty = l2; solver = liblinear
Support Vector Machine (SVM)	Grid Search	ROC AUC	5-fold CV	Exhaustive (all grid combinations)	C: [0.1, 1, 10, 100]; kernel: [linear, rbf]; gamma: [scale, auto, 0.01, 0.1, 1]	C = 1; kernel = rbf; gamma = scale
Naïve Bayes (NB)	Grid Search	ROC AUC	5-fold CV	Exhaustive (all grid combinations)	var_smoothing: [1e-12, 1e-11, 1e-10, 1e-9, 1e-8, 1e-7]	var_smoothing = 1e-9
AdaBoost	Grid Search	ROC AUC	5-fold CV	Exhaustive (all grid combinations)	n_estimators: [50, 100, 200]; learning_rate: [0.01, 0.1, 0.5, 1.0]	n_estimators = 100; learning_rate = 0.5
Random Forest (RF)	Randomized Search	ROC AUC	5-fold CV	30 iterations	n_estimators: [100, 200, 300, 500]; max_depth: [None, 5, 10, 20]; min_samples_split: [2, 5, 10]; min_samples_leaf: [1, 2, 4]; max_features: [sqrt, log2, None]	n_estimators = 300; max_depth = 10; min_samples_split = 5; min_samples_leaf = 2; max_features = sqrt
Decision Tree (DT)	Randomized Search	ROC AUC	5-fold CV	30 iterations	max_depth: [None, 3, 5, 10, 20]; min_samples_split: [2, 5, 10]; min_samples_leaf: [1, 2, 4]; criterion: [gini, entropy]	max_depth = 5; min_samples_split = 10; min_samples_leaf = 4; criterion = gini
K-Nearest Neighbors (KNN)	Randomized Search	ROC AUC	5-fold CV	30 iterations	n_neighbors: [3, 5, 7, 9, 11, 15]; weights: [uniform,	n_neighbors = 7; weights = distance; metric = minkowski

					distance]; metric: [euclidean, manhattan, minkowski]	
Extra Trees (ET)	Randomized Search	ROC AUC	5-fold CV	30 iterations	n_estimators: [100, 200, 300, 500]; max_depth: [None, 5, 10, 20]; min_samples_split: [2, 5, 10]; max_features: [sqrt, log2, None]	n_estimators = 300; max_depth = None; min_samples_split = 5; max_features = sqrt
Linear Discriminant Analysis (LDA)	Grid Search	ROC AUC	5-fold CV	Exhaustive (compatible grid combinations)	solver: [svd, lsqr, eigen]; shrinkage: [None, auto]	solver = lsqr; shrinkage = auto
Gradient Boosting (GB)	Randomized Search	ROC AUC	5-fold CV	30 iterations	learning_rate: [0.01, 0.05, 0.1, 0.2]; max_depth: [2, 3, 5]; n_estimators: [100, 200, 300]; subsample: [0.6, 0.8, 1.0]; min_samples_split: [2, 5, 10]	learning_rate = 0.05; max_depth = 3; n_estimators = 200; subsample = 0.8; min_samples_split = 5
Bottleneck Network	Manual Grid Search + Early Stopping	Validation loss	Held-out validation set (n = 47)	18 configurations	Bottleneck layer size: [8, 16, 32]; dropout rate: [0.2, 0.3, 0.5]; learning rate: [1e-2, 1e-3]; batch size: [16]; epochs: up to 100; early stopping patience: 10	Bottleneck layer size = 16; dropout = 0.3; learning rate = 1e-3; batch size = 16; epochs = 100 max; patience = 10
Very Deep Network	Keras Tuner – Random Search	Validation loss	Held-out validation set (n = 47)	30 trials	Number of layers: [3, 4, 5, 6]; neurons per layer: [32, 64, 128, 256]; dropout rate: [0.1, 0.2, 0.3, 0.5]; L1/L2 regularization: [None, L2(1e-4), L2(1e-3)]; learning rate:	4 layers; neurons = [128, 64, 32, 16]; dropout = 0.3; L2 = 1e-4; learning rate = 1e-3

					[1e-2, 1e-3, 1e-4]	
Pyramid Network	Keras Tuner – Random Search	Validation loss	Held-out validation set (n = 47)	30 trials	Number of layers: [3, 4, 5]; layer-width schedule: [256-128-64, 128-64-32, 64-32-16]; dropout rate: [0.1, 0.2, 0.3, 0.5]; learning rate: [1e-2, 1e-3, 1e-4]	3 layers; width schedule = 256-128-64; dropout = 0.3; learning rate = 1e-3
Wide Network	Keras Tuner – Random Search	Validation loss	Held-out validation set (n = 47)	30 trials	Neurons per layer: [128, 256, 512]; number of layers: [2, 3]; dropout rate: [0.2, 0.3, 0.5]; L2 regularization: [1e-5, 1e-4, 1e-3]; learning rate: [1e-2, 1e-3, 1e-4]	256 neurons/layer; 2 layers; dropout = 0.3; L2 = 1e-4; learning rate = 1e-3

Note: For the eight classical machine learning classifiers tuned via Grid or Randomized Search (LR, SVM, NB, AdaBoost, RF, DT, KNN, ET), all searches used 5-fold cross-validation with ROC AUC as the optimization criterion, as described in Section 3.4.1. For the four deep learning architectures, hyperparameter selection used a held-out validation set (n = 47) with early stopping based on validation loss; the Very Deep Network additionally used the Keras Tuner Random Search framework over 30 trials, as reported in the main text.

Table S3 Completed TRIPOD-AI checklist with manuscript page and line references.

TRIPOD-AI domain	Reporting element	Location in revised manuscript	Notes
**Title**	Identification of the study as a clinical prediction/classification modeling study using machine learning	p. 1, lines 3–9	Title states explainable machine learning approach
**Abstract**	Objectives, design, participants, methods, main results, and conclusion	p. 1, lines 26–64	Abstract explicitly distinguishes full-feature and restricted frameworks
**Background and rationale**	Clinical context and rationale for the study	p. 2, lines 19–59; p. 3, lines 6–27	Includes burden of T2DM, bariatric surgery, and need for interpretable ML

**Objectives**	Clear statement of study aims and research questions	p. 3, lines 6–18; p. 3, line 45 to p. 4, line 7	Includes RQ1–RQ3 and dual-framework aim
**Source of data**	Study design and source of data	p. 5, lines 10–20; p. 7, lines 6–10	Retrospective cross-sectional study
**Study setting**	Clinical setting and institutional context	p. 5, lines 41–50	King Fahad Specialist Hospital, tertiary referral center
**Participants**	Eligibility criteria and analytic sample	p. 5, lines 21–30; p. 5, lines 45–50	Includes screening, exclusions, and final cohort
**Outcome definition**	Definition of T2DM remission and assessment timing	p. 5, lines 15–16; p. 9, lines 38–58	ADA-based remission definition
**Predictors**	Description of predictor variables	p. 7, lines 15–28; p. 7, lines 34–49; p. 8, lines 6–63	Clinical, anthropometric, temporal, and behavioral variables
**Predictor timing**	Timing of predictors relative to outcome	p. 6, lines 15–34; p. 8, lines 57–63	Explicit distinction between preoperative and contemporaneous predictors
**Sample size**	Number of participants and outcome distribution	p. 5, lines 21–30; p. 7, lines 18–19	n = 233; 147 remission, 86 non-remission
**Missing data**	Missing-data handling and exclusions	p. 5, lines 21–30; p. 9, lines 6–25	Complete-case analysis described
**Comparison of included/excluded patients**	Assessment of possible selection bias	p. 5, lines 26–30; p. 9, lines 18–25	Included vs excluded patients compared
**Model types**	Candidate ML and DL models described	p. 5, lines 51–57; p. 10, lines 10–36; p. 11, lines 23–35	Fourteen classifiers listed
**Analytical frameworks**	Definition of Framework A and Framework B	p. 6, lines 13–52	Clear distinction between classification and prediction
**Model development**	Training strategy and feature-set use	p. 11, lines 46–52; p. 12, lines 6–22	Includes fold structure and model fitting setup
**Hyperparameter strategy**	Hyperparameter tuning approach	p. 11, lines 55–65; p. 12, lines 6–59	Grid search, randomized search, Keras tuner
**Overfitting safeguards**	Regularization, dropout, early stopping, validation monitoring	p. 11, lines 36–41; p. 12, lines 31–57	Explicitly added in revision
**Internal validation**	Hold-out evaluation and cross-validation	p. 9, lines 26–35; p. 14, lines 9–18; p. 15, lines 18–23	Test set and 10-fold validation both described
**Class imbalance handling**	Strategy used across models	p. 11, lines 57–62	Class weighting and weighted loss functions
**Performance metrics**	Discrimination and general evaluation metrics	p. 12, line 62 to p. 13, line 32	Accuracy, precision, recall, F1-score, ROC AUC, SHAP
**Calibration assessment**	Calibration plot and discussion of calibration limitations	p. 25, lines 13–21; p. 25, lines 30–31	Visual calibration only; quantitative indices not computed

**Explainability**	SHAP / TreeSHAP interpretability approach	p. 18, lines 43–54; p. 19, lines 19–40; p. 23, lines 26–36	Global and local explanation approaches
**Interpretation of SHAP**	Clarification that SHAP reflects model attribution, not causality	p. 19, lines 37–40; p. 20, lines 6–23	Explicit caution against biological over-interpretation
**Main performance results**	Best-performing models within each framework	p. 16, lines 20–34; p. 18, lines 23–40; p. 26, lines 27–38	Bottleneck Network for full-feature framework; Extra Trees for preoperative framework
**Comparative clinical benchmarking**	Comparison with DiaRem and Ad-DiaRem	p. 24, lines 10–18; p. 25, lines 13–21; p. 26, lines 27–38; p. 27, lines 7–9	Reconstructed exploratory comparison
**Model stability**	Caution regarding model ranking instability	p. 19, lines 8–15; p. 33, lines 28–38	Ranking sensitivity acknowledged
**SHAP stability**	Bootstrap assessment and caution on SHAP ranking stability	p. 19, lines 8–15; p. 22, lines 19–23	200 bootstrap resamples reported
**Clinical utility**	Decision-curve analysis and threshold interpretation	p. 25, lines 34–46; p. 26, lines 13–18	Threshold range and net benefit discussed
**Limitations**	Study limitations and future directions	p. 32, lines 22–53; p. 33, lines 6–48	External validation, cross-sectional design, sample size, missing data, calibration
**Generalizability**	Transportability and need for external validation	p. 29, lines 17–20; p. 32, lines 25–34; p. 33, lines 44–48	Single-center limitation explicitly discussed
**Conclusion**	Final interpretation and clinical caution	p. 34, lines 8–41	Balanced conclusion with implementation caution
**Funding / ethics / availability / conflicts**	Administrative reporting items	p. 35, lines 12–27	Funding, IRB, consent, availability, conflicts

Table S4. Bootstrap 95% confidence intervals for accuracy, precision, recall, F1-score, and Brier score for the 10 cross-validated machine learning models (10-fold stratified cross-validation, n = 233; out-of-fold predictions; bootstrap resampling as used for the AUC estimates in Figure 6).

Model	Accuracy (95% CI)	Precision (95% CI)	Recall (95% CI)	F1-score (95% CI)	Brier Score (95% CI)
Random Forest (RF)	0.727 (0.669–0.783)	0.615 (0.505–0.718)	0.533 (0.402–0.661)	0.571 (0.459–0.675)	0.214 (0.185–0.247)
Extra Trees (ET)	0.708 (0.648–0.765)	0.600 (0.492–0.701)	0.545 (0.413–0.670)	0.571 (0.460–0.673)	0.210 (0.181–0.241)
Gradient Boosting (GB)	0.704 (0.643–0.760)	0.667 (0.547–0.770)	0.444 (0.319–0.574)	0.533 (0.418–0.641)	0.217 (0.188–0.249)

Support Vector Machine (SVM)	0.652 (0.590–0.712)	0.571 (0.452–0.683)	0.444 (0.319–0.574)	0.500 (0.388–0.610)	0.235 (0.203–0.269)
Logistic Regression (LR)	0.691 (0.629–0.749)	0.641 (0.522–0.746)	0.610 (0.486–0.726)	0.625 (0.514–0.729)	0.221 (0.191–0.253)
AdaBoost	0.607 (0.543–0.669)	0.500 (0.377–0.623)	0.278 (0.172–0.398)	0.357 (0.245–0.474)	0.241 (0.209–0.276)
K-Nearest Neighbors (KNN)	0.661 (0.599–0.720)	0.571 (0.449–0.683)	0.500 (0.376–0.624)	0.533 (0.420–0.641)	0.229 (0.197–0.262)
Naïve Bayes (NB)	0.674 (0.612–0.733)	0.579 (0.458–0.691)	0.528 (0.402–0.651)	0.552 (0.439–0.661)	0.227 (0.196–0.260)
Decision Tree (DT)	0.592 (0.528–0.654)	0.667 (0.286–1.000)	0.111 (0.031–0.238)	0.200 (0.067–0.359)	0.246 (0.213–0.281)
Linear Discriminant Analysis (LDA)	0.682 (0.620–0.741)	0.621 (0.501–0.731)	0.563 (0.438–0.683)	0.591 (0.478–0.697)	0.223 (0.192–0.256)

Table S5. Single-variable ablation analysis of the Bottleneck Network after sequential removal of individual outcome-proximal predictors.

**Variable removed**	**AUC**	**ΔAUC vs 0.889**
None (full-feature Bottleneck Network)	0.889	0.000
Current HbA1c	0.856	0.033
OHA status	0.865	0.024
Post-surgery BMI	0.847	0.042

%WR	0.834	0.055
Post-surgery weight	0.858	0.031
%EWL	0.872	0.017
%TWL	0.874	0.015
Meal frequency	0.878	0.011
Snack frequency	0.881	0.008

Table S6. Cumulative ablation analysis of the Bottleneck Network after progressive removal of outcome-proximal predictors.

Reduced model	Variables removed	AUC	ΔAUC vs 0.889
Model 0	None	0.889	0.000
Model 1	Current HbA1c + OHA status	0.830	0.059
Model 2	Model 1 + post-surgery BMI	0.802	0.087
Model 3	Model 2 + %WR	0.775	0.114
Model 4	Model 3 + post-surgery weight	0.768	0.121
Model 5	Model 4 + %EWL + %TWL	0.763	0.126
Model 6	Model 5 + meal frequency + snack frequency	0.760	0.129
Lower reference	Preoperative-only best ML benchmark	0.707	0.182
